# SHANK1 facilitates non-small cell lung cancer processes through modulating the ubiquitination of Klotho by interacting with MDM2

**DOI:** 10.1038/s41419-022-04860-3

**Published:** 2022-04-25

**Authors:** Bo Chen, Hongye Zhao, Min Li, Quan She, Wen Liu, Jiayi Zhang, Weihong Zhao, Shuhong Huang, Jianqing Wu

**Affiliations:** 1 Department of Geriatrics, Jiangsu Provincial Key Laboratory of Geriatrics, First Affiliated Hospital, Nanjing Medical University, 210029 Nanjing, China; 2 Institute of Basic Medicine, Shandong Provincial Hospital, Shandong First Medical University and Shandong Academy of Medical Sciences, 250067 Jinan, China

**Keywords:** Non-small-cell lung cancer, Ubiquitylation

## Abstract

SH3 and multiple ankyrin repeat domains 1 (SHANK1) is a scaffold protein, plays an important role in the normal function of neuron system. It has recently been shown to be a potential oncogene. In the present study, we report that the expression of SHANK1 is upregulated in non-small cell lung cancer (NSCLC), and is correlated with clinic pathological characteristics of NSCLC. Moreover, SHANK1 overexpression enhances the proliferation, migration and invasion of NSCLC cells. Mouse cell-derived xenograft model also confirmed the effects of SHANK1 on tumor growth in vivo. Furthermore, we found that SHANK1 increases the protein degradation of Klotho (KL), an important tumor suppressor, through ubiquitination-dependent pathway. In particular, we report discovery of KL as a SHANK1-interacting protein that acts as a new substate of the E3 ubiquitin ligase MDM2. SHANK1 can form a complex with KL and MDM2 and enhance the interaction between KL and MDM2. Our findings reveal an important oncogenic role and mechanism of SHANK1, suggesting SHANK1 can be a potential therapeutic target in NSCLC.

## Introduction

Lung cancer is one of the most common malignant tumors in the world, with a high mortality rate. Non-small cell lung cancer (NSCLC) is the most common histological type of lung cancer, accounting for 85–90% of all lung cancer cases. A large number of patients with NSCLC were diagnosed at an advanced stage. Although a variety of treatments, including surgery, have achieved great progress, the 5-year survival rate is still <15% [1]. More research on the molecular mechanism underlying the proliferation, migration and invasion of NSCLC will undoubtedly provide useful guidance for the diagnosis and treatment of lung cancer.

SH3 and multiple ankyrin repeat domains (SHANK) 1–3 belong to a scaffold protein family. SHANK proteins are mainly localized in the postsynaptic densities (PSD) domain of neuronal excitatory synapses in the central nervous system. At present, it is believed that the SHANK family is essential for the normal function of the nervous system [2–4]. An association between cases of autism spectrum disorder (ASD) and mutations in the genes SH3 and multiple ankyrin repeat domains protein 1 (SHANK1), SHANK2 and SHANK3 was reported in several large-scale genomic studies. SHANK scaffolding proteins affects the structure and function of neural circuits and alters behaviors [5]. SHANK1 plays a critical role in cognition, for instance, and its dysregulation causes serious impairments in learning and cognitive assessments [4]. The SHANK1 protein is also expressed in other organs in the periphery, though its functions are largely unknown. Recently, it was reported that SHANK1 was highly expressed in colon cancer, and that such patients were more likely to receive a more severe prognosis. The knockdown of SHANK1 inhibited viability and induced apoptosis in colon cancer cell lines through AKT/mTOR signaling pathways. Methylation alteration at this SHANK1 CpG island is used as a biomarker for risk and diagnosis of chronic lymphocytic leukemia. Those reports suggest that SHANK1 may be a new oncogene present in cancers [6, 7]. SHANK2 is a recently reported Hippo pathway regulator, amplified in human cancer and potently promotes cancer [8]. SHANK3 mutations have been recognized as a genetic risk factor for ASD [9–11]. However, there is little known about how SHANKs are involved in tumor processes in lung cancers.

Klotho (KL) is a potent tumor suppressor in many malignancies, including colorectal, glioma, melanoma, and ovarian cancers [12–16]. Several signal mechanisms have been reported to be involved in the tumor suppressor activity of KL, including the IGF-1, FGF, and Wnt/β-catenin pathways [13], while the detailed working mode of KL in cancer is still a matter of controversy [17–19]. We previously reported the role of KL in the pathogenesis of NSCLC, showing that KL inhibits NSCLC proliferation and motility, and that it triggers apoptosis by negatively modulating IGF-1/insulin signaling and the Wnt signaling pathway [20–23]. Moreover, we found overexpression of KL inhibits the HELF fibroblasts SASP-related protumoral effects on NSCLC cells. Even ample studies demonstrated the important tumor suppressor roles of KL, there is currently only limited information regarding the potential molecular mechanisms by which KL is regulated. Some researchers reported that KL levels are epigenetically downregulated in cancer [14, 17–19]. Recently, we found that Rab8 is involved in the membrane localization of KL and the abnormal regulation of Wnt signaling pathway caused by KL [24]. However, the functions of other KL interacting proteins still need further investigation. Based on immunoprecipitation and mass spectrometry detection, we found that SHANK1 is a new KL binding protein.

In this study, we report that SHANK1 is upregulated in NSCLC, and contributes to the proliferation, migration and invasion of NSCLC cells through forming a complex with KL and the E3 ubiquitin ligase MDM2, and modulating the MDM2-dependent degradation of KL.

## Results

### SHANK1 is upregulated in NSCLC

**Fig. 1:**
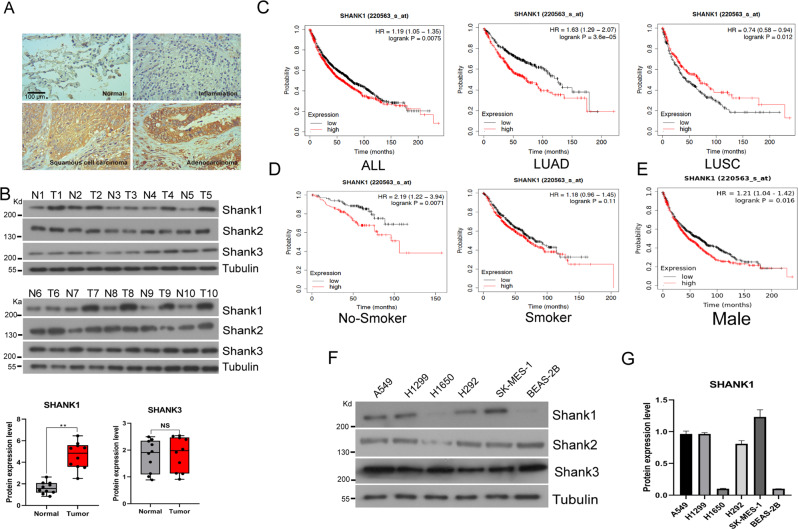
SHANK1 is upregulated in NSCLC samples and causes cell proliferation in NSCLC cell lines. **A** SHANK1 expression was examined by immunohistochemical staining of NSCLC tissue samples. **B** Protein expression of SHANK1, SHANK2 and SHANK3 in NSCLC samples, as measured by WB, relative to that of Tubulin. T tumor. N normal. **C** Prognostic value of SHANK1 in NSCLC patients were assessed by using the Kaplan–Meier (KM) plotter integrative data analysis tool (http://www.kmplot.com). The desired Affymetrix ID is valid: 220563_at (SHANK1). Survival curves are plotted for all NSCLC patients (ALL). Survival curves are plotted for adenocarcinoma patients (LUAD). Survival curves are plotted for squamous cell carcinoma patients (LUSC). **D**, **E** Survival curves are plotted for non-smoker, smoker, and male patients. **F**, **G** Protein expression of SHANK1, SHANK2 and SHANK3 in different lung cancer cell lines.

To identify the expression of SHANK1 in NSCLC tissue samples, we examined the protein level of SHANK1 in NSCLC tissues and their corresponding adjacent normal lung tissues by immunohistochemical analysis. Fifty out of 100 NSCLC tumor tissues showed abnormally high expression of SHANK1, significantly higher than that of their corresponding adjacent normal lung tissues (Fig. 1A). Further, we examined protein expression in 10 pairs of NSCLC tissues (not the same tissues with IHC) and corresponding adjacent normal lung tissues by western blot analysis. The results show that SHANK1 was overexpressed in tumor tissue samples. Compared with corresponding normal lung tissue samples, the expression levels of SHANK2 and SHANK3 were not changed (Fig. 1B). Using Kaplan–Meier analyses, we differentiated a high-risk group from low-risk based on gene expression (log-rank test, *p* < 0.01). The overall survival rate in NSCLC patients with high SHANK1 expression was lower than those with low SHANK1 expression (Fig. 1C). Base on abovementioned results, we found SHANK1 was obviously high-expressed in NSCLC especially in LUAD patients and closely related to tumor prognosis, we mainly focused on the role of SHANK1 in LUAD cells in our follow-up study. Moreover, we found the correlation was only present in the non-smoker and male groups (Fig. 1D, E). Further, we tested SHANK1, SHANK2, and SHANK3 protein expression in multiple NSCLC cell lines (A549, H1299, H1650, H292, SK-MES-1) compared with BEAS-2B cells (epithelial cells isolated from normal human bronchial epithelium from autopsy of non-cancerous individuals), and found SHANK1 expression was elevated in most NSCLC cell lines (Fig. 1F, G). Then, we focused on whether SHANK1 played an important role in NSCLC.

### Knockdown of SHANK1 suppresses migration and invasion, and induces apoptosis of NSCLC cells

**Fig. 2:**
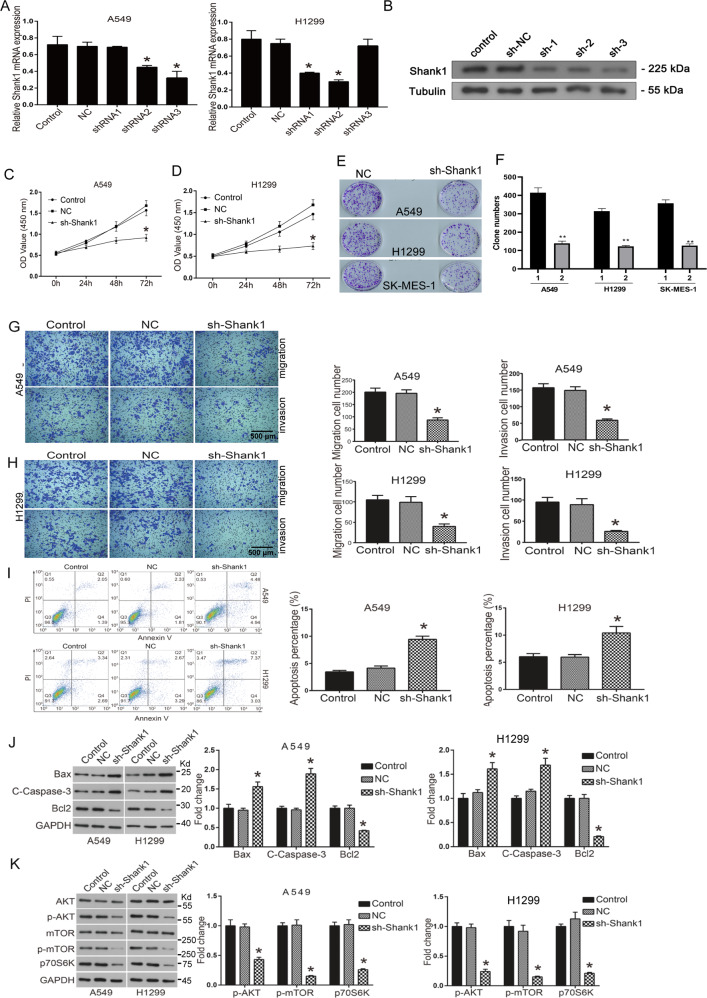
Knockdown of SHANK1 inhibits the migration and invasion, and it increases the apoptosis of human lung cancer cell lines. **A** A549 and H1299 cells were transfected with SHANK1 shRNAs, followed by qRT-PCR assays to detect knockdown efficiency of siRNAs. **B** A549 cells were transfected with SHANK1 shRNAs, followed by WB assays to detect knockdown efficiency of siRNAs. **C**, **D** CCK8 assay showed that SHANK1 knockdown inhibited cell proliferation in A549 and H1299. **E**, **F** Clone formation assay showed that SHANK1 knockdown inhibited cell growth in A549, H1299, and SK-MES-1. **G** Transwell was performed to detect the migration and invasion of A549 cells. The number of migratory and invasive A549 cells was counted. **H** Transwell was performed to detect the migration and invasion of H1299 cells. The number of migratory and invasive H1299 cells was counted. *p < 0.05. **I** The apoptosis of A549 and H1299 cells was detected by flow cytometry. The column diagram shows the apoptosis cell number. **J** The apoptosis-related protein levels were detected by western blot. The column diagram shows the related expression levels of target genes. **K** The PI3K-AKT-related protein levels were detected by western blot. The column diagram shows the related expression levels of target genes. *p < 0.05.

We applied the interruption approaches using specific shRNAs to block SHANK1 expression, and selected the most effective sequence shRNA2# for subsequent experiments (Fig. 2A, B). By CCK8 assays, we observed that depletion of SHANK1 (sh-SHANK1) remarkably decreased the OD value at 48 and 72 h in both A549 and H1299 cells (*p* < 0.05, Fig. 2C, D). We further used clone formation assay to investigate cell proliferation of A549 and H1299 cells. To get a prolonged knockdown effect, we constructed the stable knockdown cell lines carrying sh-SHANK1 sequences. As shown in Fig. 2E, F, the number of clones in the sh-SHANK1 group decreased to ~60% of the shNC group in three cell lines.

To investigate whether SHANK1 expression was involved in cell migration and invasion, transwell assays were carried out in A549 and H1299 cells transfected with sh-SHANK1 or shRNA (NC). As predicted, the knockdown of SHANK1 dramatically reduced the numbers of migrated and invasive A549 as well as H1299 cells (Fig. 2G, H) (*p* < 0.01). These observations suggest that SHANK1 was a positive regulator of NSCLC motility. To determine whether the growth-inhibitory effects of sh-SHANK1 results from cell death, we assessed cell apoptosis by flow cytometry. As shown in Fig. 2I, the proportion of apoptotic cells in the control shRNA (NC) or sh-SHANK1-transfected A549 cells was 4.17% and 9.42%, respectively, suggesting that the observed decrease in the number of living cells upon sh-SHANK1-transfection was indeed caused by cell death (*p* < 0.01). The same result was found in H1299 cells (Fig. 2I).

To further investigate the molecular mechanism of SHANK1 regulating cell apoptosis cell apoptosis, we detected the expression of apoptosis-associated proteins. As shown in Fig. 2J, the silencing of SHANK1 significantly increased the expression of Bax and Active-Caspase-3, and it decreased the expression of anti-apoptotic protein Bcl-2. The data suggests that the downregulation of SHANK1 promoted cell apoptosis through the regulation of apoptosis-associated proteins. In order to determine the mechanism underlying the function of SHANK1 on NSCLC cells, we investigated the PI3K-AKT/mTOR signaling pathway. The PI3K-AKT/mTOR signaling pathway is reported to play a central role in regulating tumor cell proliferation, cell cycle, apoptosis, and movements. As shown in Fig. 2K, the phosphorylated levels of AKT and mTOR in A549 and H1299 cells were significantly downregulated when silencing SHANK1 (*p* < 0.05). These data suggest that the downregulation of SHANK1 deactivated the AKT/mTOR signaling pathway in NSCLC cells.

### SHANK1 overexpression reduces the protein expression of KL in a dose-dependent manner

**Fig. 3:**
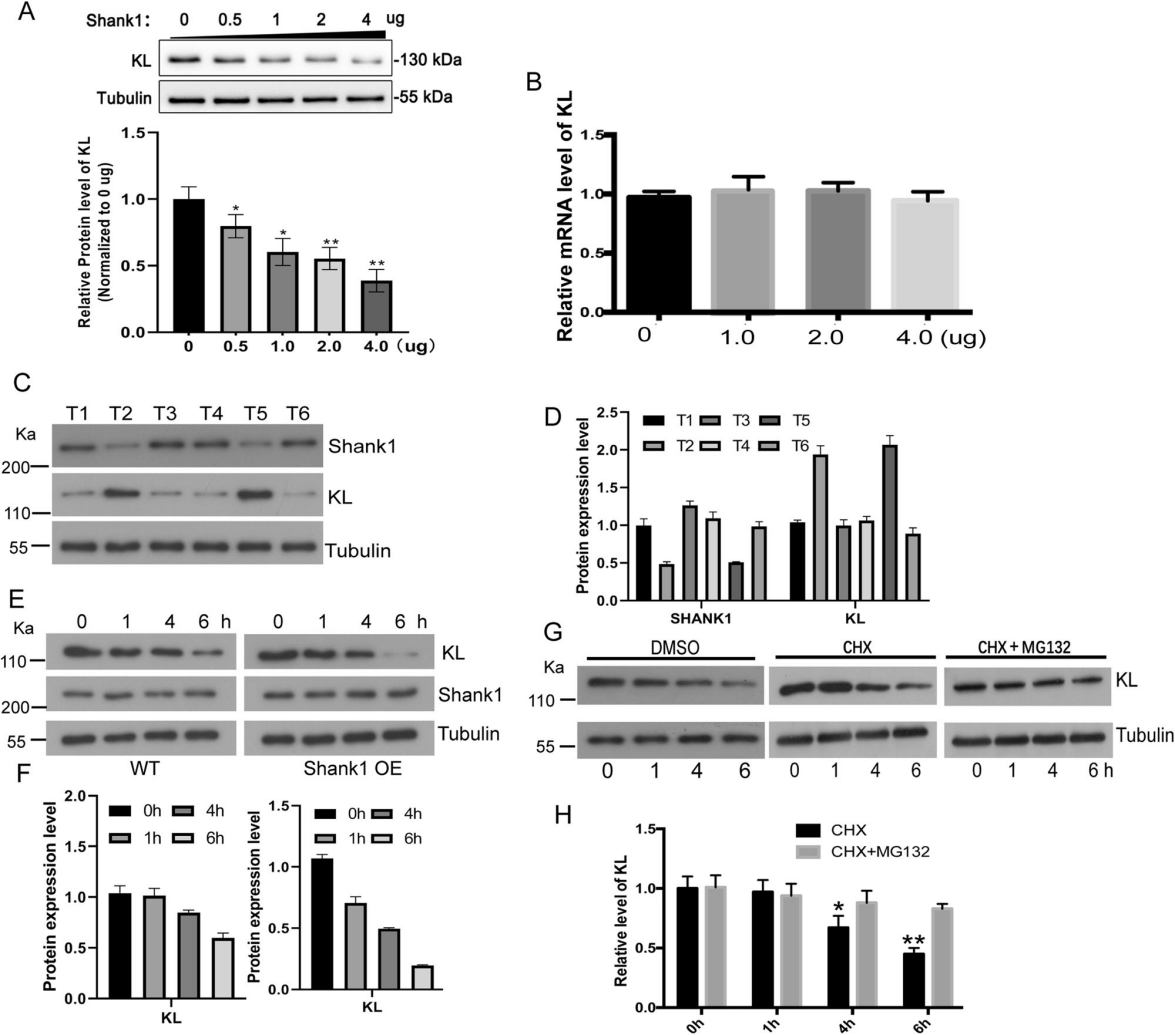
SHANK1 overexpression reduces the protein expression of KL in a dose-dependent manner. **A** A549 cells were transfected with SHANK1, and relative protein levels of KL were detected by western blotting assay. Data shown are the mean ± SEM of three separate experiments (*p < 0.05, compared with the control group (0 ng), one-way ANOVA). **B** A549 cells were transfected with SHANK1, and relative mRNA levels of KL were detected by qRT-PCR. **C**, **D** Protein expression of SHANK1 and KL in NSCLC samples, as measured by WB, relative to that of Tubulin. T tumor. N normal. **E**, **F** A549 cells were transfected with empty vector or SHANK1 for 48 h and then serum-starved for the indicated times at 37 °C in the presence of cycloheximide (CHX, 20 μg/ml). KL was detected by anti-KL immunoblotting. Data shown are the mean ± SEM of three separate experiments (*p < 0.05, analysis of variance followed by post hoc tests). **G**, **H** A549 cells were transfected with SHANK1 vector for 48 h and then serum-starved for the indicated times at 37 °C in the presence of DMSO(1/1000), cycloheximide (CHX, 20 μg/ml) and MG132 (10 μM). KL was detected by anti-KL immunoblotting. Data shown are the mean ± SEM of three separate experiments (*p < 0.05, analysis of variance followed by post hoc tests).

Recent studies suggest that SHANK1 proteins are essential to developmentally regulating the activation of Akt and correlated intracellular pathways crucial for mammalian postnatal brain development and synaptic plasticity, while the detailed molecular function of SHANK1 is largely unknown. We reported previously that KL serves as a tumor suppressor gene in NSCLC, and that it inhibits the process of NSCLC [15, 20, 23–26]. We wanted to investigate whether SHANK1 plays a role in the expression or function of KL. Interestingly, we found that SHANK1 overexpression reduces the protein expression of KL in a dose-dependent manner (Fig. 3A), while the mRNA level of KL was not affected significantly (Fig. 3B). To further explore the relationship between the expression of SHANK1 and KL, we performed a correlation study on SHANK1 and KL protein expression in six NSCLC patients using western blot analysis. As shown in Fig. 3C, D, four tumor tissues presented high expression of SHANK1, but with low expression of KL. In contrast, two tumor tissues presented low expression of SHANK1, but high expression of KL. Overall, SHANK1 expression was significant in its negative correlation with KL expression in lung cancer tissue. The decrease in KL protein levels could be caused by the reduction of new KL synthesis or by the increase of KL degradation. To determine which process was involved in the SHANK1 regulated decrease in KL protein levels, A549 cells were preincubated with the protein biosynthesis inhibitor (cycloheximide, CHX) for indicated times before detecting the time cause of KL expression, and the protein level of KL was detected by western blot. As shown in Fig. 3E, F, SHANK1 overexpression increased the degradation of KL. As shown in Fig. 3G, H, when SHANK1 is overexpressed, the degradation rate of KL protein was not affected by protein biosynthesis inhibitor, but blocked by proteasome inhibitor, MG132. That is to say, MG132 could rescue the degradation caused by SHANK1 overexpression. Our results indicate that SHANK1 may regulate KL protein levels via degradative pathways as opposed to biosynthetic pathways.

### SHANK1 interacts with KL, and increases the ubiquitination of KL

**Fig. 4:**
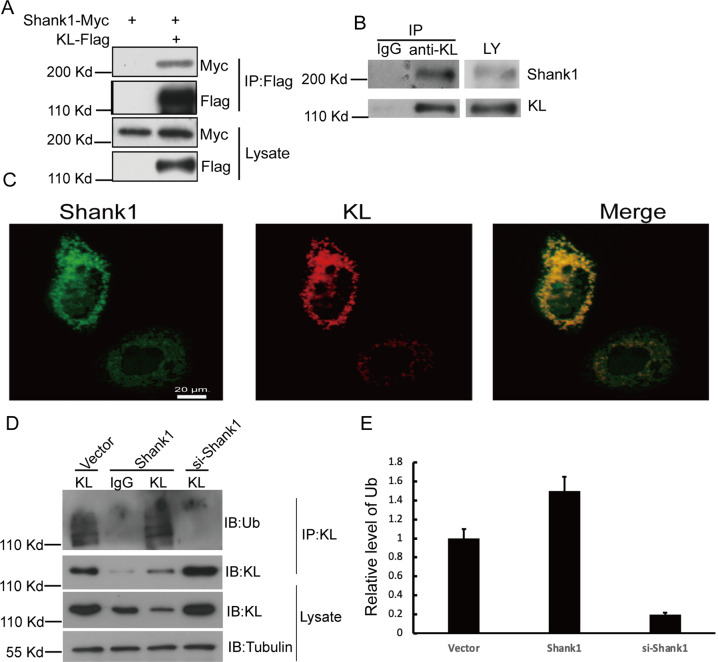
SHANK1 regulates ubiquitination of KL by interacting with KL. **A** A549 cells were transfected with SHANK1-myc and Flag-KL constructs and lysed by TNE buffer, followed by immunoprecipitation with anti-FLAG antibody. Western blotting analysis was performed. **B** Endogenous association of KL and SHANK1 in adult rat lung tissue was detected by co-immunoprecipitation. **C** Subcellular co-localization of endogenous KL and SHANK1 in A549 cells visualized by confocal microscopy. Staining was carried out with rabbit anti-SHANK1 (green) and mouse anti-KL (red) antibodies. **D** The ubiquitination of KL was detected by IP and western blotting with Ub antibody. **E** The relative Ub levels in (**D**) are shown (relative to vector expression level, the error bars indicate SEM for three experiments, **p < 0.01, determined by student’s *t* test).

To define molecular basis of how KL protein level is subjected to SHANK1 regulation, we performed co-immunoprecipitation experiments [24] and found that SHANK1 appeared in KL’s IP precipitate. Therefore, SHANK1 is a potential KL interacting protein. We wanted to confirm that there is an association between SHANK1 and KL. In transient transfection and co-immunoprecipitation experiments, we found that SHANK1 shared an intriguing interaction with KL in A549 cells (Fig. 4A). Moreover, to exclude the possibility of ectopic overexpression artifact, the endogenous SHANK1 and KL interaction was examined in A549 cells (Fig. 4B). Then, we used SHANK1 and KL antibodies to examine the localization of endogenous SHANK1 and KL. The results indicated that SHANK1 was partially colocalized with KL (Fig. 4C). Furthermore, as shown in Fig. 4D, E, SHANK1 remarkably increased the ubiquitination of KL, while SHANK1 knocking-down decreased the ubiquitination of KL.

### MDM2 interacts with KL, and increases the ubiquitination of KL

**Fig. 5:**
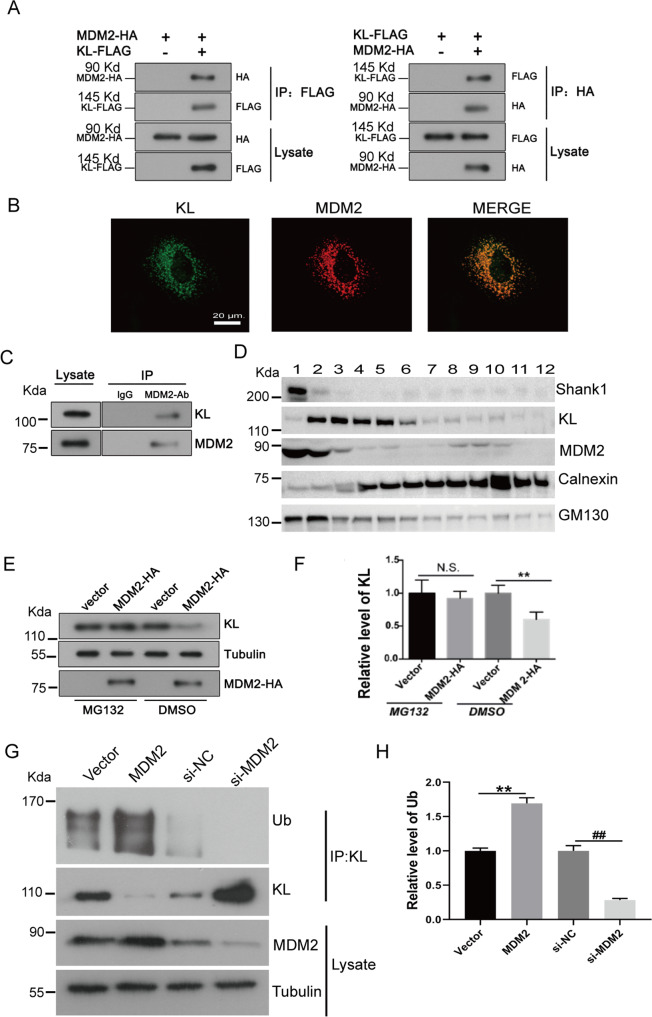
MDM2 regulates ubiquitination of KL by interacting with KL. **A** Left: Co-immunoprecipitation of MDM2 with KL. Lysates from A549 cells transfected with MDM2-HA and Flag-KL constructs were immunoprecipitated with anti-Flag antibodies. Immunoblotting analysis was performed to detect immunoprecipitated proteins. Right: Lysates from A549 cells transfected with MDM2-HA and Flag-KL constructs were immunoprecipitated with anti-HA antibodies. Immunoblotting analysis was performed to detect immunoprecipitated proteins. **B** Subcellular co-localization of endogenous KL and MDM2 in A549 cells visualized by confocal microscopy. Staining was carried out with rabbit anti-KL (green) and mouse anti-MDM2 (red) antibodies. **C** Endogenous KL associates with MDM2. Rat lung lysates were subjected to immunoprecipitation with mouse anti-MDM2 or rabbit anti-KL antibodies. **D** Post-nuclear supernatants of A549 cells were fractioned on an isopycnic 15–55% (w/w) linear sucrose gradient, and equal aliquots of the final equilibrium gradient fractions were immunoblotted using antibodies against GM130, calnexin and KL, Shank1 and MDM2. **E** A549 cells were transfected with vector or MDM2 constructs. Next, they were treated with/without MG132 and lysed. The protein level of KL was detected by western blotting. **F** The relative protein levels in (**E**) were shown (relative to vector expression level, the error bars indicate SEM for three experiments, **p < 0.01, determined by student’s *t* test). **G** A549 cells were transfected with either vector (pCDNA3.1), pCDNA3.1-MDM2, or si-NC, si-MDM2 for 48 h, and lysed by TNE buffer. The ubiquitination of KL was detected by IP and western blotting with Ub antibody. **H** The relative Ub levels in (**G**) are shown (relative to vector or siNC expression level, the error bars indicate SEM for three experiments, **p < 0.01, determined by student’s *t* test).

All the results above suggest that SHANK1 may play a role in modulating the ubiquitination and degradation of KL. We wanted to determine the potential E3 ubiquitin ligase of KL. To address this problem, we reviewed previous IP-MS result, and found MDM2 was a potential interacting protein of KL. MDM2 is one of the most well-validated negative regulators of p53. As an E3 ubiquitin ligase, it can downregulate p53 activity through modulating poly-ubiquitylation of p53 and targeting p53 for proteasome-mediated degradation, exporting p53 out of the nucleus, or direct binding to p53 to block transactivation of key targets [27, 28]. Upregulated MDM2 was detected in many malignancies such as lung cancer, breast cancer, liver cancer, esophagogastric cancer, colorectal cancer, and etc. [29]. MDM2 can also enhance p21 degradation. In addition, MDM2 is associated with various protein molecules such as E2F, p19 (Arf), and Ras-mitogen-activated protein kinase (MAPK) [29]. We wanted to explore whether MDM2 could enhance KL degradation through modulating its ubiquitination. First, we examined whether MDM2 could form a complex with KL. We transfected Flag-KL with C-terminal MDM2-HA in HEK-293 cells. After immunoprecipitation of KL with FLAG antibodies, an association of KL and MDM2 was observed and assessed by immunoblotting analysis using HA antibodies (Fig. 5A). The complex between the Flag-KL and MDM2-HA was also detected after immunoprecipitation of HA and immunoblotting with anti-Flag antibodies. We used MDM2 and KL antibodies to examine the localization of endogenous MDM2 and KL by immunocytochemistry and confocal microscopy. The results indicated that MDM2 was partially colocalized with KL (Fig. 5B). To determine whether this interaction occurs under physiological conditions, we performed endogenous KL/MDM2 co-immunoprecipitation assays from A549 cells. Lysates were immunoprecipitated with anti-MDM2 antibodies, and we found that KL co-immunoprecipitated with MDM2 under the endogenous level (Fig. 5C). To further confirm that Klotho was co-located with MDM2 in physiological condition, we performed a sucrose density gradient centrifugation assay to separate organelles in A549 cells and used GM130 and calnexin to identify Golgi- and ER-enriched fractions, respectively (Fig. 5D). We observed that both KL and MDM2 were present in fractions 2–3. Also, SHANK1 was partially present in fraction 2. Thus, we confirmed that MDM2/KL could form a complex both in vitro and in vivo. Further, we found that MDM2 could decrease KL expression, and this effect could be blocked by MG132 (a specific proteasome inhibitor) (Fig. 5E, F). Additionally, we examined whether MDM2 overexpression could enhance the ubiquitination of KL. The constructor expressing MDM2 (tagged with HA) or shRNA of MDM2 was transfected into A549 cells, respectively. We found MDM2 overexpression could increase the ubiquitination of KL significantly compared with empty vector control, while the si-MDM2 could decrease the ubiquitination of KL (Fig. 5G, H). All the results suggest that MDM2 could modulate the ubiquitination of KL, and might serve as an E3 ubiquitin ligase of KL, and SHANK1 could modulate their interaction.

### SHANK1, KL, and MDM2 can form a complex

**Fig. 6:**
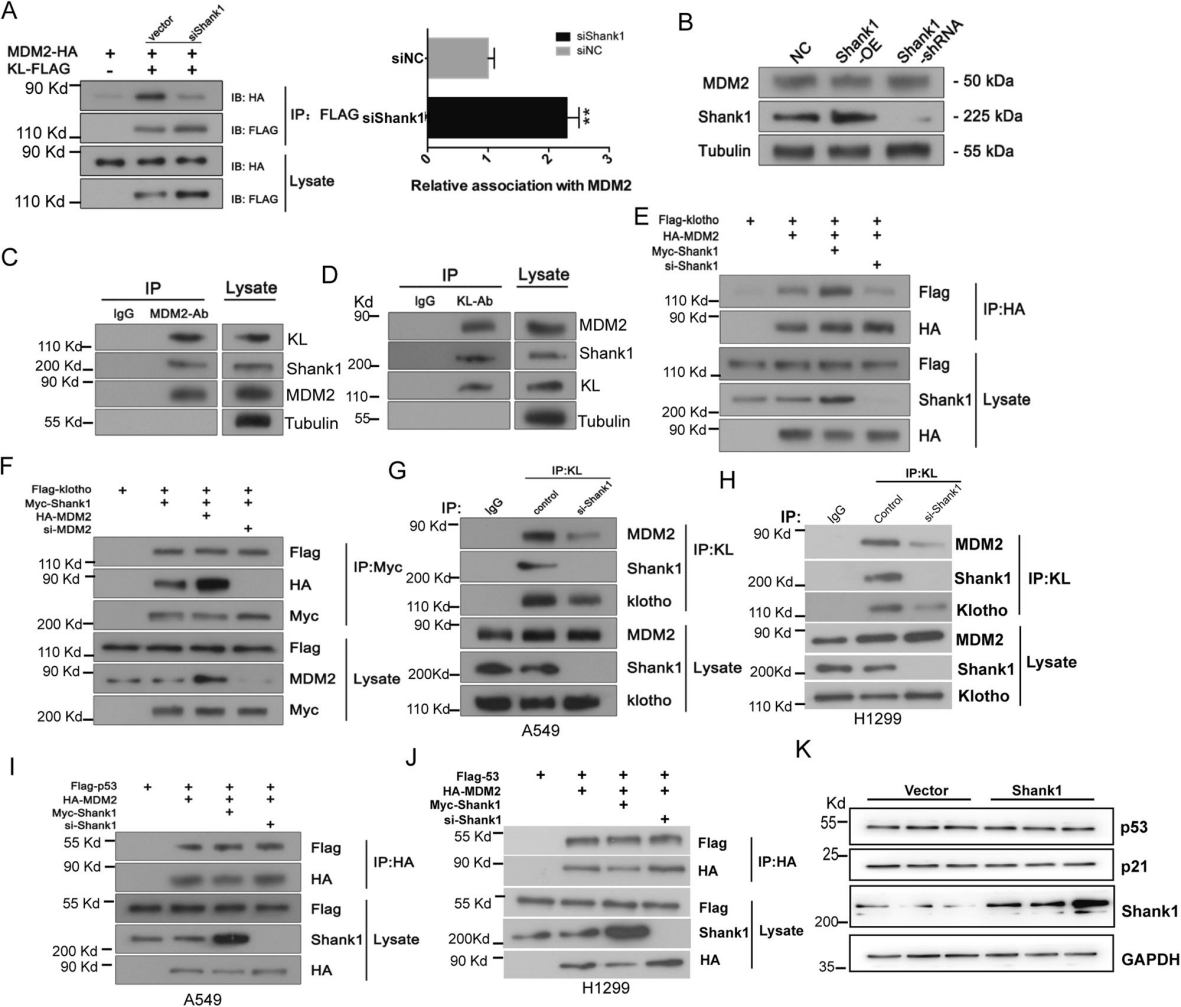
Ternary complex formation of KL, MDM2, and SHANK1 in vitro and in vivo. **A** Co-immunoprecipitation of MDM2 with KL. Lysates from A549 cells transfected with MDM2-HA, KL-Flag, or sh-SHANK1 constructs were detected by immunoprecipitation and immunoblotting analysis. **B** Lysates from A549 cells transfected with SHANK1-myc or sh-SHANK1 vector were detected by immunoblotting analysis to show the protein expression of MDM2. **C**, **D** Endogenous MDM2 associates with SHANK1 and KL. A549 cell lysates were subjected to immunoprecipitation with mouse anti-MDM2 or Rabbit anti-KL antibodies, and then the protein complex was eluted. MDM2, KL, and SHANK1 were detected by immunoblotting. **E** Lysates from A549 cells co-transfected with Myc-SHANK1/or si-SHANK1. MDM2-HA and Flag-KL constructs were immunoprecipitated with anti-HA antibodies. Immunoblotting analysis was then performed to detect immunoprecipitated proteins. **F** Lysates from A549 cells co-transfected with Myc-SHANK1, MDM2-HA/or si-MDM2, and Flag-KL constructs were immunoprecipitated with anti-Myc antibodies. Next, immunoblotting analysis was performed to detect immunoprecipitated proteins. **G** Lysates from A549 cells transfected with si-SHANK1 were collected, and then immunoblotting analysis was performed by KL antibody to detect immunoprecipitated proteins. **H** Lysates from H1299 cells transfected with si-SHANK1 were collected, and then immunoblotting analysis was performed by KL antibody to detect immunoprecipitated proteins. **I** Lysates from A549 cells co-transfected with Myc-SHANK1/or si-SHANK1, MDM2-HA, and Flag-p53 constructs were immunoprecipitated with anti-HA antibodies. **J** Lysates from H1299 cells co-transfected with Myc-SHANK1/or si-SHANK1, MDM2-HA, and Flag-p53 constructs were immunoprecipitated with anti-HA antibodies. **K** A549 cells were transfected with vector or SHANK1-myc constructs and lysed by TNE buffer, followed by immunoblotting with anti-p53 and p21 antibodies.

To examine whether SHANK1 could affect the interaction between KL and MDM2. We found that SHANK1 knockdown could decrease the association between KL and MDM2 significantly (Fig. 6A). However, the expression of MDM2 was not affected by SHANK1 (Fig. 6B). As shown in Fig. 6C, after immunoprecipitation of MDM2 with specific antibodies, an association of KL with SHANK1 was also observed as assessed by immunoblotting. This association could also be found in the immunoprecipitation of KL (Fig. 6D). SHANK1 overexpression could increase the interaction between KL and MDM2, and the silence of SHANK1 could decrease the interaction (Fig. 6E), while the expression level of MDM2 could not affect the association between KL and SHANK1 (Fig. 6F). Endogenous MDM2/KL co-immunoprecipitation assays were also performed. We also found that SHANK1 co-immunoprecipitated with KL under the endogenous level, and that silencing SHANK1 could debilitate the interaction between MDM2 and KL in A549 cells (Fig. 6G). We also repeated this experiment in H1299 cells (without p53 expression), and got the same results (Fig. 6H). Given that MDM2 is an important E3 ubiquitin ligase of p53, we wanted to detect whether SHANK1 could also modulate the interaction between p53 and MDM2. We performed COIP assay in both A549 and H1299 cells, and the results were shown in Fig. 6I, J. Intriguingly, we found SHANK1 didn’t affect the association between p53 and MDM2. Moreover, SHANK1 did not affect the protein level of p53 and p21 (Fig. 6K). In all, our results indicate that SHANK1, KL, and MDM2 could form a complex specifically, and that SHANK1 affected the formation of the complex and modulated the protein level of KL.

### Knockdown of SHANK1 inhibits tumor growth in mouse model

**Fig. 7:**
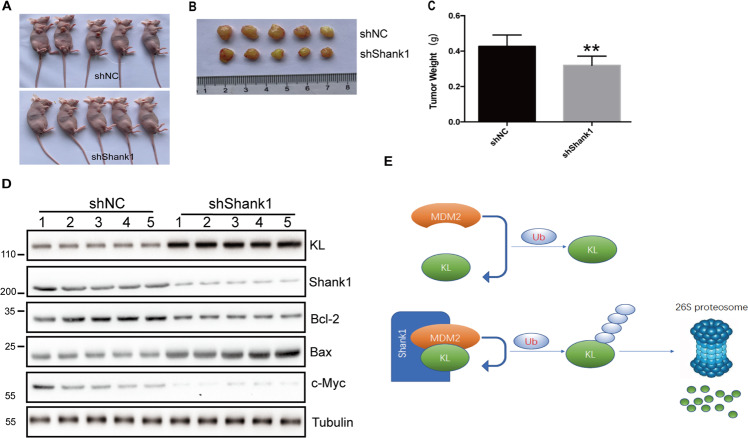
SHANK1 knocking-down decrease NSCLC tumorigenesis in vivo. **A**, **B** The nude mice were inoculated with either A549 cells stably expressing shNC or sh-SHANK1, and the xenograft tumors were photographed. **C** Tumor weight at the end point (3 weeks). ^**^p < 0.01, compared with NC group student’s *t* test. **D** The expression of KL, Shank1, Bcl-2 and c-Myc of tumor xenograft was examined by immunoblotting. **E** Simplified model depicting the complex of KL, MDM2, and SHANK1.

To further confirm the reliability of abovementioned research results in vitro, we injected xenografted A549 cells expressing shRNA of SHANK1 into BALB/c nude mice. After 3 weeks, the mice were sacrificed, and the tumor weights were further examined. As shown, mice who received A549-sh-SHANK1 injection had smaller tumors than the control group (Fig. 7A–C). Moreover, we detected the protein level of KL, Shank1, Bcl-2 and Bax (apoptosis-related protein) and c-Myc (proliferation related protein) (Fig. 7D). The results indicate that Shank1 knocking down could inhibit the proliferation of cancer cells.

## Discussion

Tumor suppressor capabilities of KL have been implicated in numerous cancer types [12, 13, 16, 24, 30, 31]. We and other research groups have found that KL is an anti-aging gene but has a lack of expression in lung cancer, and that it plays an important role in the development of lung cancer, drug resistance, etc. [14, 15, 20–24, 32]. Many physiological activities, signaling pathways, and proteins are involved the process. Recently, we found that rab8 is involved in the membrane localization of KL and the abnormal regulation of Wnt signaling pathway caused by KL [32]. However, the functions of other KL interacting proteins still need further investigation to discover valuable new oncogenes that can be used to unravel the unidentified crucial mechanisms in tumorigenesis. The present study establishes the role of SHANK1 as a novel oncogene in NSCLC and reveals, for the first time, an association between KL and SHANK1 in this context.

The SHANK family of proteins are known as anchoring/scaffold proteins in postsynaptic density (PSD) of excitatory synapses [33]. The demonstrated interaction of SHANK proteins with various membrane receptors and cytoskeletons suggests that they may play a central role in the assembly of the postsynaptic macro complex and in transmission of the signals to cytoskeletal molecules. SHANK proteins are relatively large proteins (200 kDa) containing multiple protein–protein interaction sites that include ankyrin repeats, SH3 domain, PDZ domain, long proline-rich region, and SAM domain. SHANK1 is abnormally and highly expressed in colon cancer; knockdown of SHANK1 inhibits the survival, proliferation, and migration of colon cancer cells through the AKT/mTOR signaling pathway. In the present study, we sought to analyze the gene expression of SHANK1 in NSCLC samples [34]. It has also been reported that the methylation alteration of SHANK1 could serve as a predictive, diagnostic, and prognostic biomarker for chronic lymphocytic leukemia [6].

In this study, we found that SHANK1 was a commonly upregulated gene in NSCLC compared to normal tissues, and that it was associated with a worse prognosis for patients. The results suggest that SHANK1 has a potential clinical significance in NSCLC. A549 and H1299 lung cancer cell lines were chosen to investigate the role of SHANK1 in cell proliferation, migration, and apoptosis. Our results show that the loss of SHANK1 significantly inhibited the proliferation and reduced migration of both cell lines. Knockdown of SHANK1 induced apoptosis in A549 and H1299 cells and affected expression of apoptosis-related proteins.

The ubiquitin-proteasome proteolysis system responsible for degrading proteins is involved in nearly all cellular processes. Dysregulation in the components of the ubiquitin system leads to a variety of diseases such as cancers. For instance, MDM2 is a famous E3 ligase for its role in p53 degradation. In this study, we report that KL underwent ubiquitination, and that SHANK1 could interact with KL and increase its MDM2-dependent ubiquitination. Furthermore, we found that MDM2 serves as a potential E3 ligase of KL. MDM2 is able to downregulate p53 activity through modulating poly-ubiquitylation of p53 and targeting p53 for proteasome-mediated degradation, exporting p53 out of the nucleus, or by directly binding to p53 to block transactivation of key targets [27, 28]. MDM2 overexpression was also detected in many malignancies including lung cancer, breast cancer, liver cancer, esophagogastric cancer, colorectal cancer, etc. [29]. MDM2 can also enhance p21 degradation. In addition, MDM2 is associated with various protein molecules such as E2F, p19 (Arf), and Ras-mitogen-activated protein kinase (MAPK) [29]. We report the ubiquitination of KL for the first time in this study, which is also a potential new substrate for MDM2. However, it should be noted that MDM2 is also an E3 ligase for neddylation [35–38], and neddylation and ubiquitination often share the same lysine sites, so we cannot eliminate the possibility that MDM2 also regulates neddylation of KL, and the ubiquitination level of KL alteration is an indirect consequence.

Our research enriches the understanding of KL regulation on post-translational modification. Our findings could help develop new strategies to increase KL expression and maintain functional stability by inhibiting its degradation. MDM2 is a famous oncogene, and research into its functions has continued for more than 25 years. Understanding the dysregulation of MDM2 and how it functions in tumorigenesis may improve methods of diagnosis and for assessing prognosis. Our research has also increased the understanding of MDM2’s cancer promoting function, detailing how it can cause its related cell dysfunction by degrading KL.

Remarkably, our study shows that SHANK1 affects the association between KL and MDM2, also acting as an important organizer to mediate KL degradation directly and specifically (it does not affect association of p53 and MDM2) (Fig. 7E). The essential roles of SHANK1 in KL degradation suggest that the deregulation of ubiquitin system component targeting KL may be involved in tumorigenesis.

In the present study, a novel KL degradation way via the ubiquitin system was elucidated. We report that SHANK1 is an oncogene involved in cell proliferation, apoptosis, transformation, invasion, and tumor progression in NSCLC. SHANK1 bound with KL and MDM2, and downregulated KL by ubiquitin degradation, and it promoted the migration, invasion, and proliferation abilities of lung cancer cells. Our data provides novel mechanistic insights into the function of SHANK1, KL, and MDM2 in NSCLC progression.

## Materials and methods

### Antibody and reagents

Rabbit and mouse monoclonal anti-KL, rabbit anti-SHANK1, and rabbit anti-MDM2 antibodies were from Abcam (ab259265, Cambridge, UK). Mouse anti-ubiquitin antibody from Covance (838701, Princeton, NJ) or Rb anti-ubiquitin antibody from PTG (10201-2-AP, PTG, China) was used to detect ubiquitination. Rabbit and mouse anti-FLAG antibodies were from Sigma Aldrich (St Louis, MO). Rat monoclonal high affinity anti-HA antibody was from Sigma Aldrich (St Louis, MO). MG132 was from MCE Company (Ann Arbor, MI). Tissue culture media and reagents were from Invitrogen (ThermoFisher, Waltham, MA), restriction endonucleases from New England Biolabs (Danvers, MA), and all other chemicals were from Sigma Aldrich, unless otherwise specified.

### Analyses of TCGA data

In order to estimate the survival rate, we made use of the Kaplan–Meier plotter, a website tool based on The Cancer Genome Atlas database, the European Genome-phenome Archive (EGA), and Gene Expression Omnibus (GEO) (Affymetrix microarrays only). The log-rank P value and hazard ratio (HR) with 95% confidence intervals (CI) were computed and are shown on the plot. GEPIA was applied to map the survival plots based on thousands of samples from TCGA.

### Immunohistochemical assay

The tissue microarray (TMA) of NSCLC was purchased from Outdo Biotech (Shanghai, China). The TMA slices were incubated in an EDTA citrate buffer (pH 8.0), and microwaved for antigenic retrieval. Then, the slides were incubated with the primary antibody (anti-SHANK1, ab154224, Abcam) at 4 °C overnight. Normal rabbit IgG were used as negative controls to ensure specificity. Next, the slides were treated by HRP polymer conjugated secondary antibody for 30 min and developed with diamino-benzidine (DAB) solution. Nuclei were counterstained with hematoxylin. Image acquisition was performed using a Nikon camera and software.

### Constructs

Plasmids encoding HA- and myc-tagged human KL have been described previously [21, 24]. Plasmids encoding SHANK1, MDM2, and related mutants were from Youbio. Other mutations were introduced by PCR and confirmed by dideoxy-sequencing.

### Cell culture and transfection

HEK-293, A549, H1299, and H650 cells were cultured in Dulbecco’s modified eagle medium supplemented with 10% FBS and 1% penicillin-streptomycin in a humidified incubator at 37 °C and 5% CO_2_. Lipofectamine 2000 (ThermoFisher) (1:2.5 DNA: lipid) in Opti-MEM was used to transfect cells according to the manufacturer’s instructions. DNA amounts in each transfection were kept constant by the addition of an empty vector. All experiments were conducted 48 h post-transfection.

### Western blot analysis

Western blot analysis was performed for protein detection. Primary rabbit anti-human antibodies, including anti-SHANK1 (ab154224, Abcam), anti-SHANK3 (ab93607, Abcam), Bcl-2 (ab32124, 1:1000), caspase3 (ab13847, 1:1000), Bax (ab32503, 1:1000), AKT (ab8805, 1:1000), p-AKT (ab38449,1:1000), cyclin D1 (ab134175, 1:1000), P70 (ab109393, 1:1000) and glyceraldehyde 3-phosphate dehydrogenase (ab181602,1:5000) were purchased from Abcam. The relative expression of the target protein was calculated by protein/internal reference using Quantity One software.

### Colony formation assay and cell proliferation assay

NSCLC cells were seeded into six-well plates at a density of 1000 cells per well, then cultured for 14 days until the appearance of colonies was evident. The colonies were fixed in 10% formaldehyde at room temperature for 15 min, followed by staining with 1% Crystal Violet. CCK8 was utilized for the cell proliferation assay. Normal cultured NSCLC cells were trypsinized and counted to create the suspension. About 1000 cells were seeded into each well of the 96-well plate, and cells treated with 0.1% DMSO were used as the control. A total of 10 µl of CCK8 reagent was added every 24 h to detect cell vitality. Following 90 min of incubation at 37 °C, the OD value of excitation light was detected using an enzyme standard instrument at a wavelength of 450 nm. Based on the OD values, a proliferation curve was drawn.

### Immunocytochemical staining

In brief, A549 cells were fixed with 4% paraformaldehyde for 10 min, then washed with PBS for three times, permeabilized with 0.4% Triton X-100 in PBS for 10 min. After being washed in PBS for three times, the cells were incubated with blocking solution (PBS containing 10% normal goat serum) for 1 h at room temperature. After incubation with indicated primary antibodies at 4 °C overnight, cells were washed with PBS and incubated with fluorescent secondary antibodies conjugated to Alexa Fluor 488 or Alexa Fluor 594, or Cy5 for 1 h at room temperature. All the immunostained cells were observed with an LSM710 confocal microscope (Carl Zeiss).

### Flow cytometry

NSCLC cells were stained with Annexin V-fluorescein isothiocyanate (FITC) and propidium iodide (PI). Flow cytometry was utilized for the detection of cell apoptosis in accordance with the manufacturer’s protocol. Following treatment, the cells were collected via centrifugation. The cell pellets obtained from centrifugation were suspended in 500 μl of binding buffer, and then 5 μl of Annexin V-FITC and 5 μl of a PI solution were applied prior to incubation at room temperature for 15 min. A FACSCalibur instrument was used to measure Annexin V and PI staining by flow cytometry, and data analysis was performed via FlowJo software.

### Cell transwell and invasion assays

Prior to beginning the experiment, the coating buffer was prepared, consisting of 0.01 M Tris pH 8.0, 0.7% NaCl and filtered by 0.2 μm sterile filter unit. Any pipets, syringes, or containers that could come into contact with the Matrigel were chilled prior to use. Overnight, the Matrigel Matrix aliquot was thawed on ice at 4 °C, and then it diluted with serum-free 1640 medium at a ratio of 1:6. A total of 100 μl was applied to each permeable support well of the 24-well plate prior to incubation at 37 °C for 4 h. Without disturbing the layer of Matrigel, we carefully removed the permeable support membrane and added 100 μl and 600 μl of serum-free 1640 medium to the inside and outside, respectively. Next, we incubated the membrane at 37 °C for 30 min. At this point, the coated invasion chambers were ready for use. The procedure for the transwell experiment was similar to the invasion experiment, but a cubicle was not required for Matrigel processing, and the number of cells was 5000.

### Immunoprecipitation

Immunoprecipitation experiments were performed as we described previously [24]. Indicated cells (HEK-293 or A549) were scraped off plates, collected by centrifugation in phosphate-buffered saline, and resuspended in the immunoprecipitation buffer (IPB) containing 50 mM Tris. HCl, 2 mM EDTA, 250 mM NaCl, 10% (v/v) glycerol, 0.5% NP-40, 20 mM NaF, 1 mM sodium orthovanadate, and 10 mM N-ethylmaleimide. Cells were lysed at 4 °C for 1 h and centrifuged to remove the debris. The supernatant was pre-cleared by incubating with 25–30 μl of Protein G Agarose for 1 h at 4 °C. Target proteins were then immunoprecipitated by incubating the supernatant overnight at 4 °C with appropriate antibodies (1–2 μg per 60 mm Petri dish) and 20–25 μl of Protein G agarose. Beads were washed three times with IPB, and the proteins were eluted by boiling in an SDS buffer for 5 min.

### Cell fractionation analysis

A549 cells (8 × 10^7^) were collected by digesting with 0.25% trypsin and washed once with PBS and then with sucrose buffer (250 mM sucrose, 10 mM triethylamine, pH 7.4, and 1 mM EDTA). Then, the cells were homogenized on ice using ~30 strokes of a glass Dounce homogenizer in 0.5 ml of buffer (320 mM sucrose, 10 mM Hepes, pH 7.2, and protease inhibitors) and centrifuged at 500 × *g* for 2 min. The supernatant was then subjected to fractionation by centrifugation at 105,000 × *g* for 2 h in an SW60 Ti rotor (Beckman Instruments) and layered on a discontinuous sucrose density gradient (15, 30, 40, and 55%). Fractions were collected from the top (designated fraction number 1 (top) to 12 (bottom)). An aliquot of each fraction (90 μl) was mixed with protein sample buffer and assayed by immunoblotting.

### SHANK1 and MDM2 knockdown

Pre-tested SHANK1 shRNA oligonucleotides were purchased from Sigma, the target sequence of Shank1(shRNA 2#):CCA AGC TTA ATG GAT GGG ATT). The target sequence of MDM2 was 5′-AAG CTT GGC ACG CCA AAC AAA-3′. The negative control oligos encoding shRNA, which is not homologous to any known mammalian sequence, were purchased from Invitrogen. The top and bottom oligonucleotides were annealed and subcloned into the pSuper-GFP vector, which co-cistronically expressed GFP to label transfected cells.

### Animal experiments and sample collections

Four-week-old male nude mice (Male C57BL/6 and BALB/c Nude mice) were purchased from Beijing Vital River Experimental Animal Technical Co., LTD. The mice were housed in a local SPF experimental animal facility with a 12 hr light/dark cycle and constant temperature and relative humidity (to about 50%). Two weeks later (6-week-old), mice were randomly divided into two groups, a total of 2 × 10^6^ A549 shShank1 or vector cells diluted in 0.1 ml of PBS were injected into the armpit of five mice per group. After growth for another 21 days, the mice were sacrificed and isolated the tumors. The investigator was blinded to the group allocation during the experiment. All experimental procedures involving animals in this study were reviewed and approved by the Institutional Animal Care and Research Advisory Committee at Nanjing Medical University.

### Statistical analysis

Each experiment was repeated at least three times independently. All data were presented as means ± standard error of the mean carried out by SPSS 19 software or GraphPad Prism 8.0. The Student’s *t* test was employed to evaluate the significant difference between the two independent groups. The one-way analysis of variance was employed to evaluate the significant difference, when the experiment that has more than two groups, followed by Bonferroni/Dunn post hoc comparison of means with correction for multiple comparisons. In all cases, *p* < 0.05 was considered significant.

## Supplementary information

Original western blots

## Data Availability

All data supporting the findings of this study are available from the corresponding author on reasonable request.
