# Supplementary material for: SHANK1 facilitates non-small cell lung cancer processes through modulating the ubiquitination of Klotho by interacting with MDM2
Source: Cell Death Dis. 2022 Apr 25;13(4):403. doi: 10.1038/s41419-022-04860-3 (PMC9039064; doi:10.1038/s41419-022-04860-3)

Fig 1B

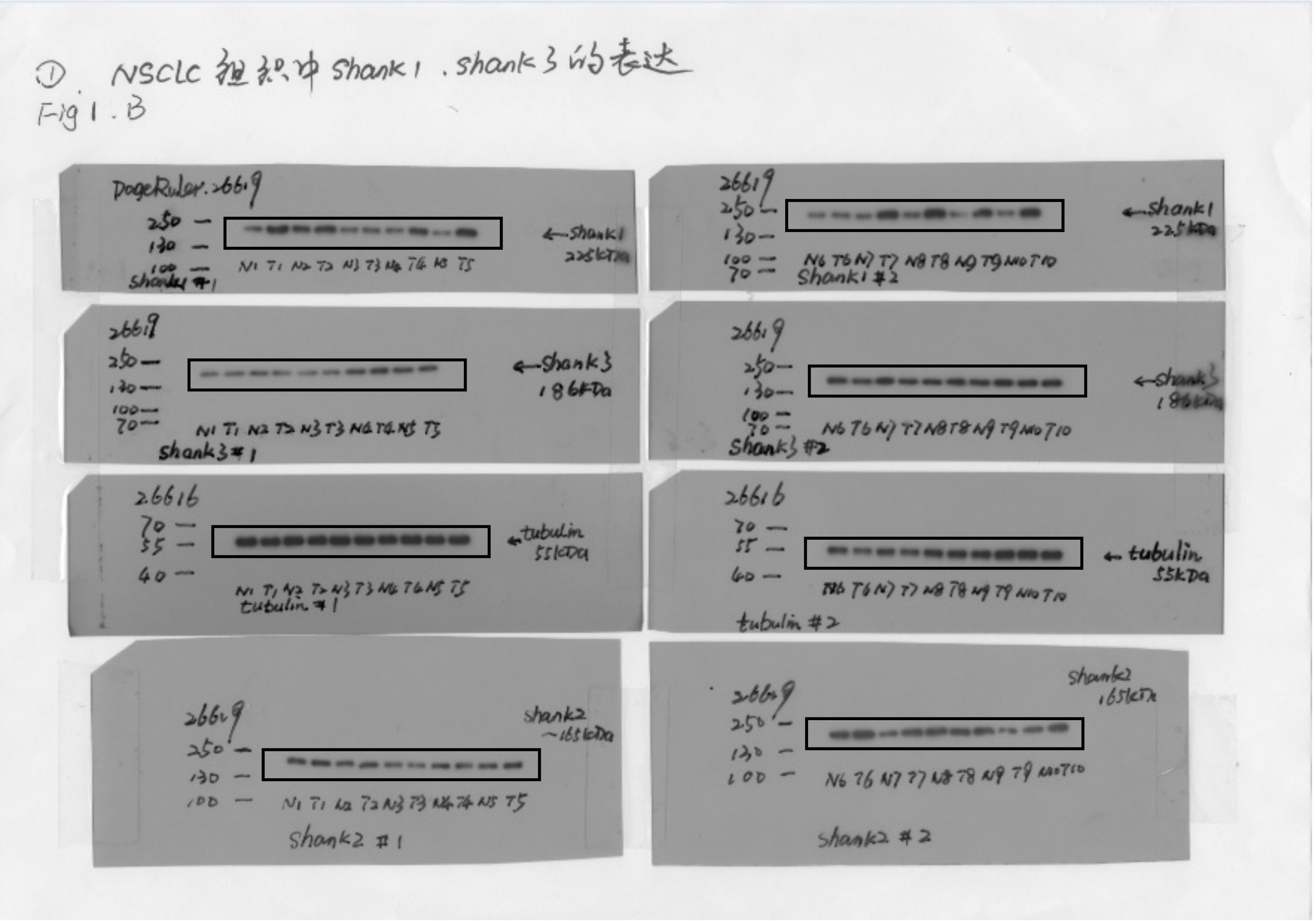
① NSCLC 组织中 shank1 . shank3 的表达
Fig 1 . B
shank1 #1
Shank1 #2
shank3 #1
Shank3 #2
tubulin #1
tubulin #2
shank2 #1
shank2 #2

Fig 1F

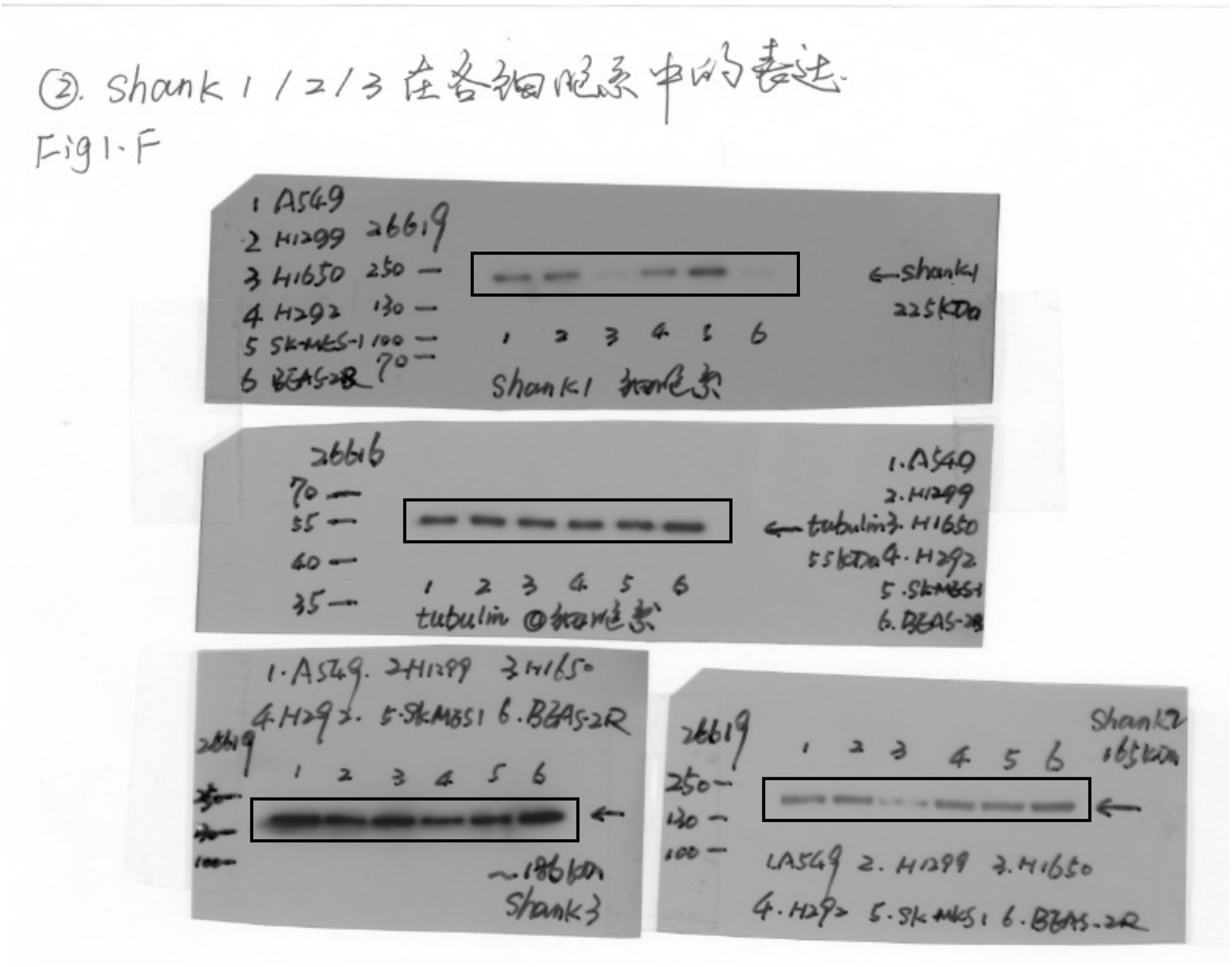
②. shank 1/2/3 在各细胞系中的表达
Fig1.F
1 A549
2 H1299
3 H1650
4 H292
5 SK-MES-1
6 BEAS-2B
250
130
100
70
1 2 3 4 5 6
shank1
225kDa
70
55
40
35
tubulin
55kDa
1 2 3 4 5 6
1. A549 2. H1299 3. H1650
4. H292 5. SKMES1 6. BEAS-2B
~186kDa
Shank3
Shank2
165kDa
250
130
100
1. A549 2. H1299 3. H1650
4. H292 5. SK-MES1 6. BEAS-2B

Fig 2B

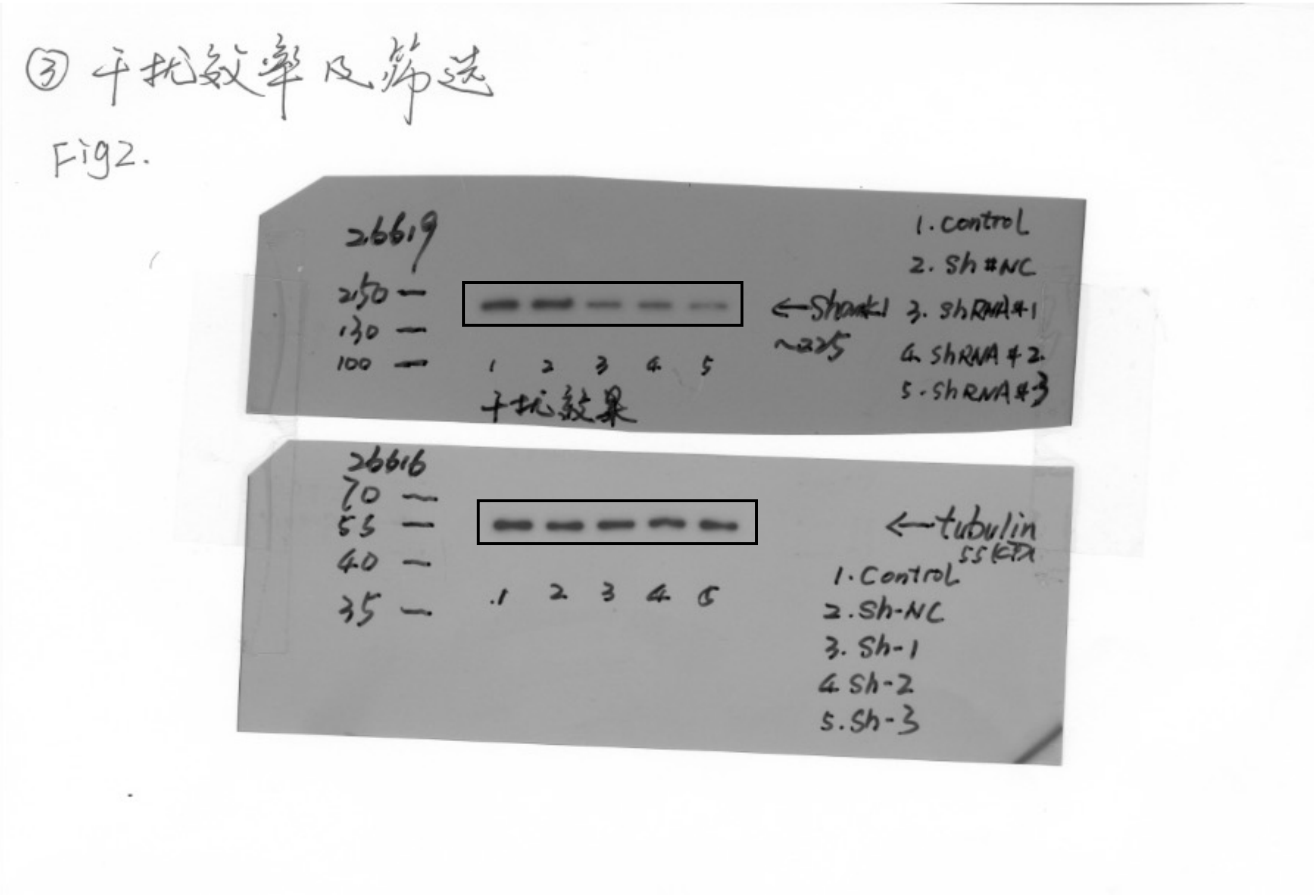
③ 干扰效率及筛选
Fig2.
26619
250
130
100
1 2 3 4 5
←Shank1 ~225
1. control
2. Sh#NC
3. shRNA#1
4. shRNA#2.
5. shRNA#3
干扰效果
26616
70
55
40
35
1 2 3 4 5
←tubulin 55KDa
1. Control
2. Sh-NC
3. Sh-1
4. Sh-2
5. Sh-3

Fig 2J 2K

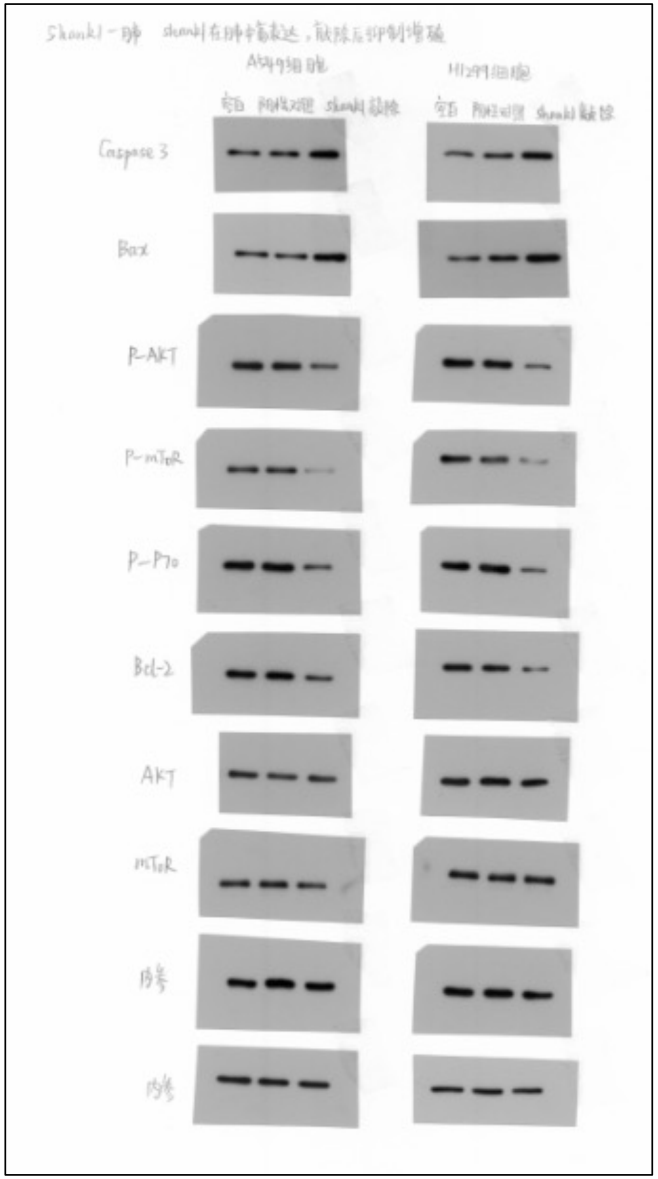
A549细胞
H1299细胞
Caspase 3
Bax
P-AKT
P-mToR
P-P70
Bcl-2
AKT
mToR
内参
内参

Fig 3A

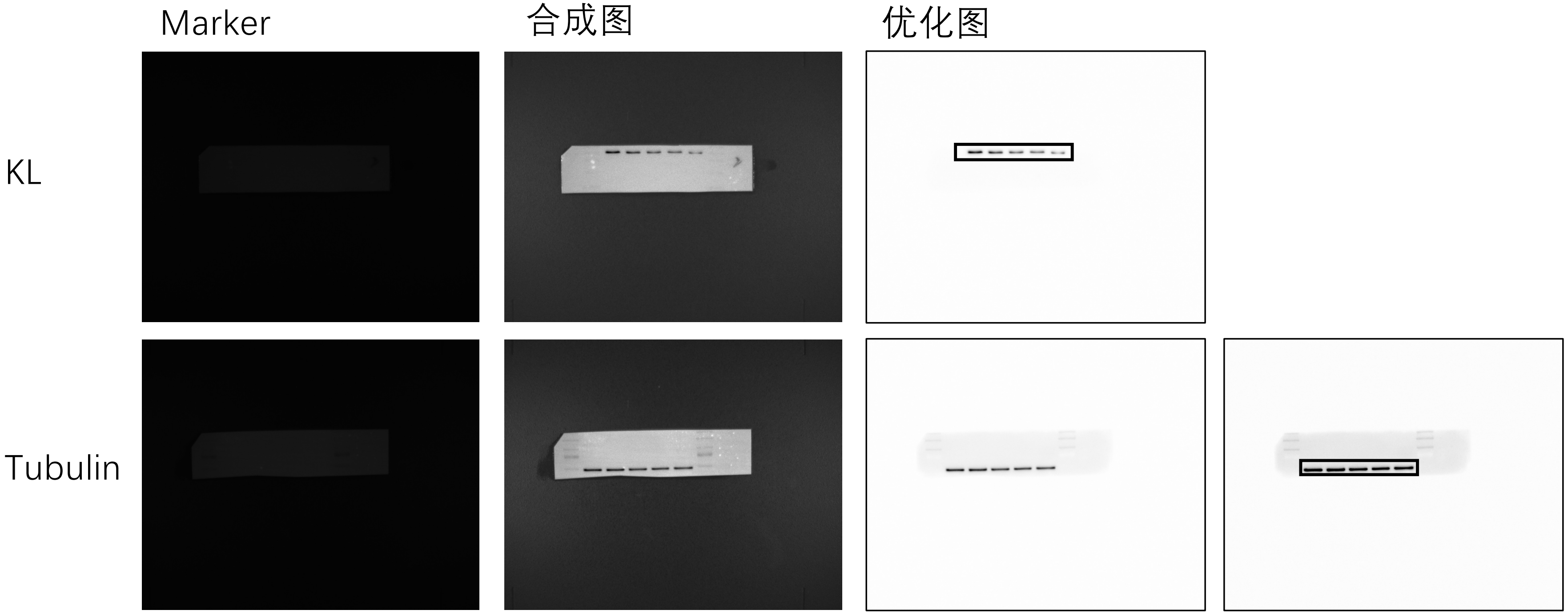

Fig 3C

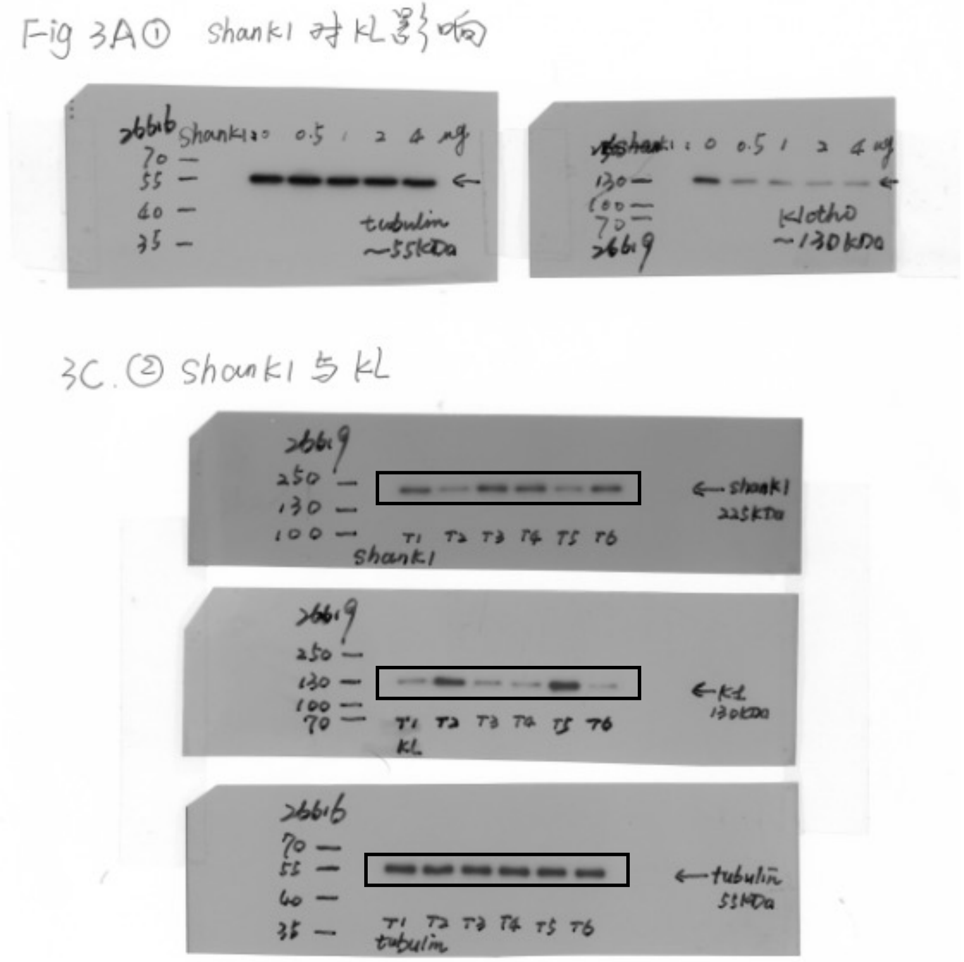

Fig 3E

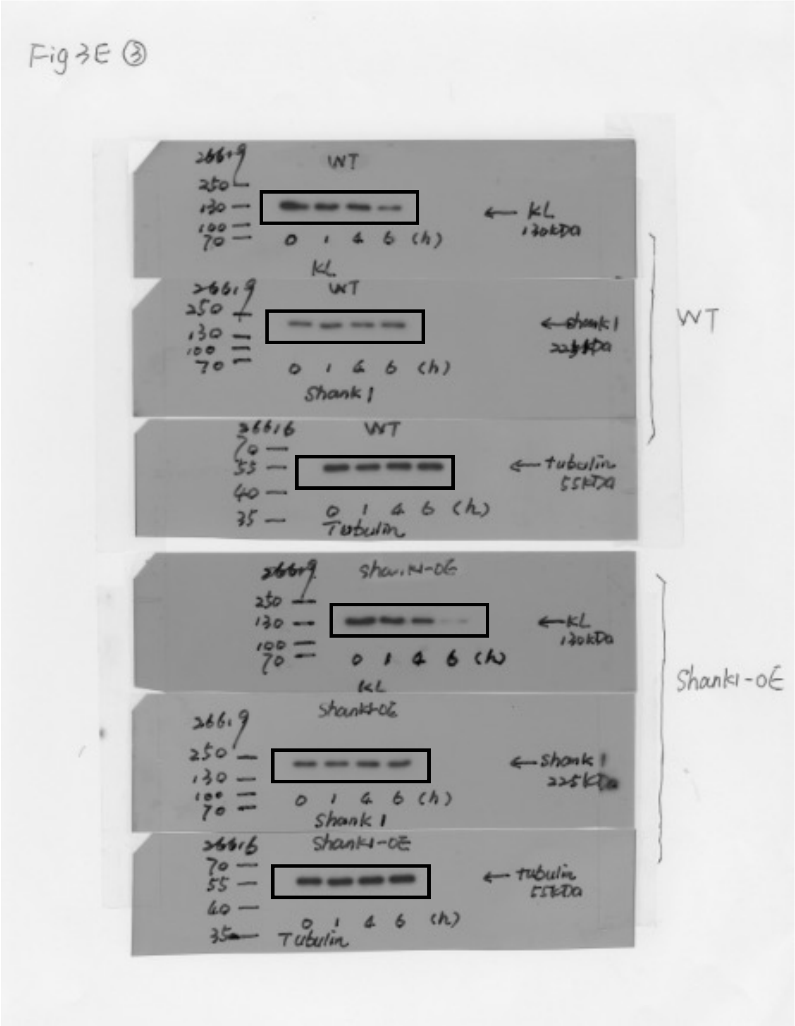
Fig 3E ③
WT
← KL
130kDa
0 1 4 6 (h)
← shank1
0 1 4 6 (h)
Shank1
← tubulin
55kDa
0 1 4 6 (h)
Tubulin
WT
← KL
130kDa
0 1 4 6 (h)
← shank1
225kDa
0 1 4 6 (h)
Shank1
Shank1-OE
← tubulin
55kDa
0 1 4 6 (h)
Tubulin
Shank1-OE

Fig 3G

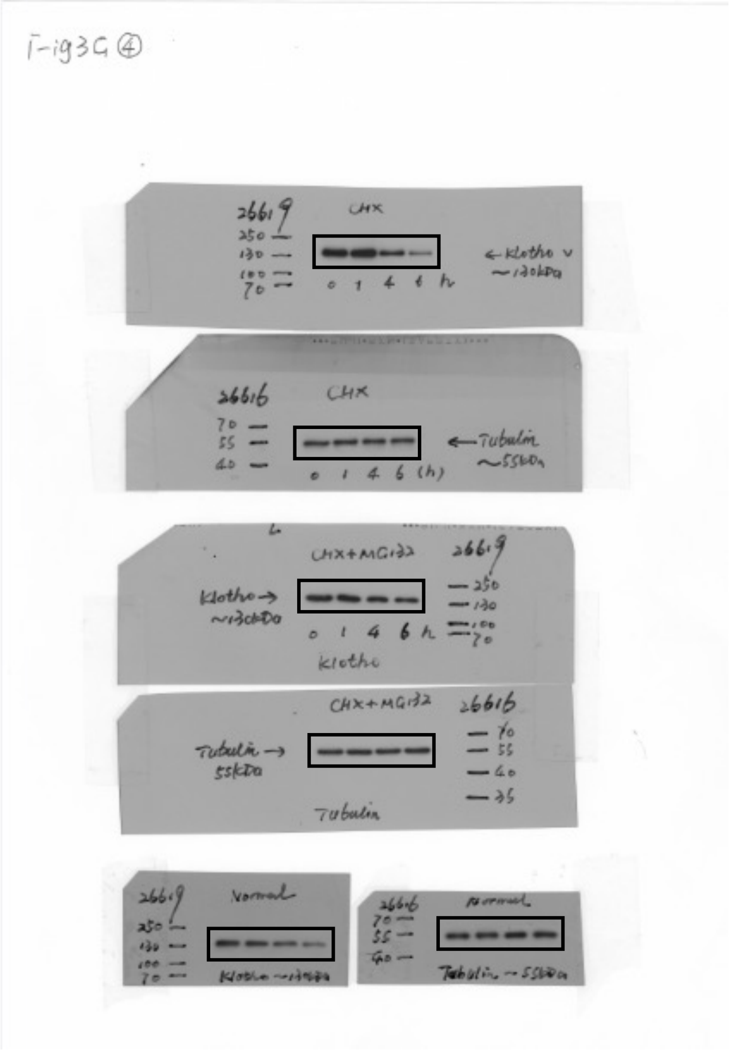
Fig3G ④
26619
CHX
← Klotho
~130kDa
0 1 4 6 h
26616
CHX
← Tubulin
~55kDa
0 1 4 6 (h)
CHX+MG132
Klotho →
~130kDa
Tubulin →
55kDa
Normal

Fig 4A， B

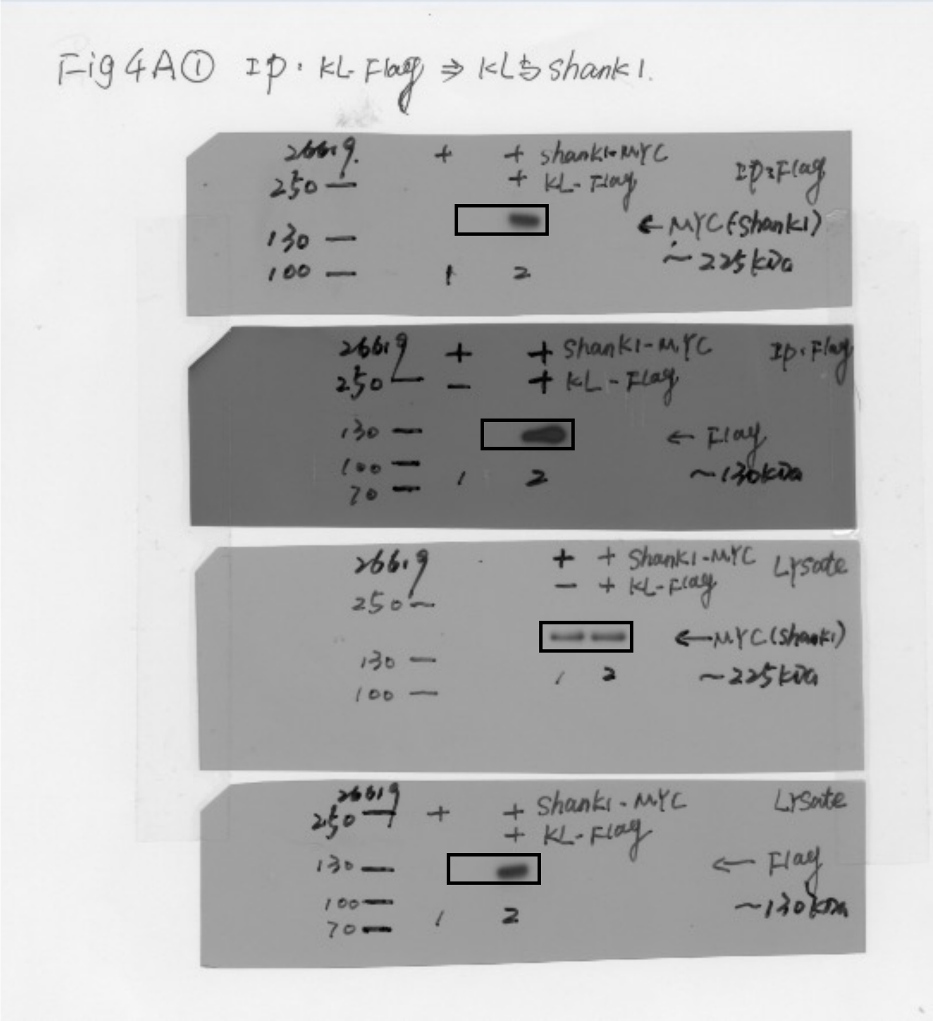
Fig 4A① IP: KL-Flag ⇒ KL与shank1.
26619
250
130
100
70
shank1-MYC
KL-Flag
IP: Flag
←MYC(shank1)
~225kDa
←Flag
~130kDa
Lysate

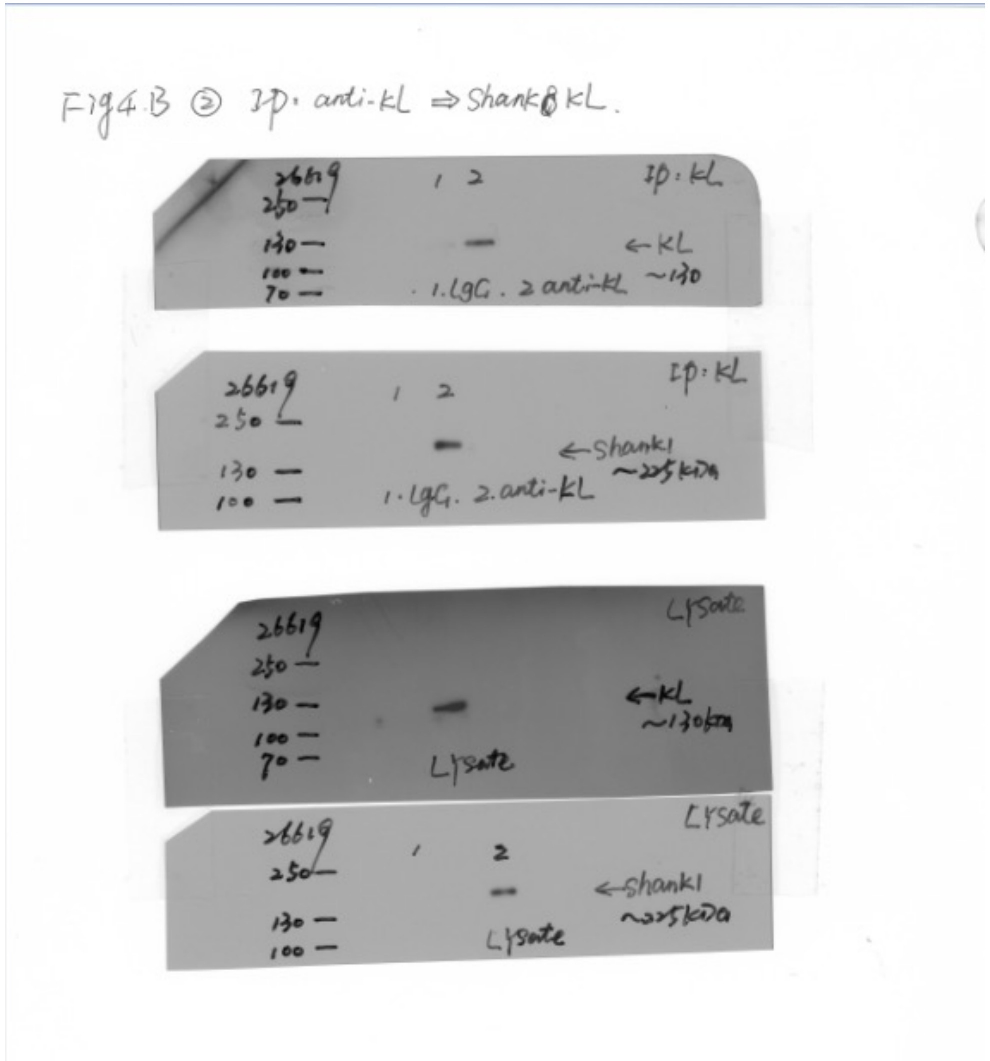
Fig4.B ② IP: anti-KL ⇒ Shank与KL.
26619
250
130
100
70
IP: KL
←KL
~130
1. IgG. 2 anti-KL
←shank1
~225kDa
Lysate
~130kDa

Fig 4D

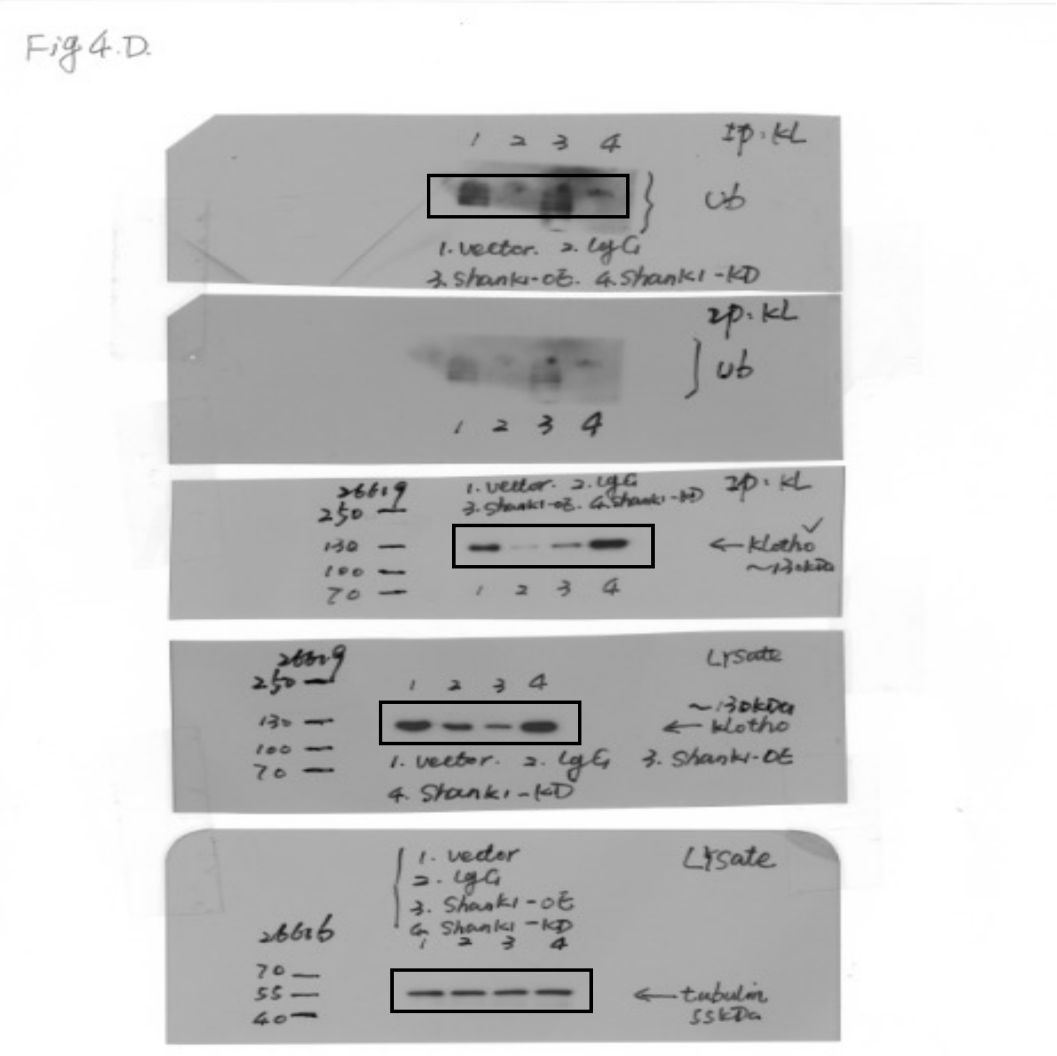
Fig 4.D.
IP: KL
ub
1. vector. 2. IgG
3. Shank1-OE. 4. Shank1-KD
IP: KL
ub
IP: KL
klotho
~130kDa
Lysate
~130kDa
klotho
1. vector. 2. IgG 3. Shank1-OE
4. Shank1-KD
Lysate
1. vector
2. IgG
3. Shank1-OE
4. Shank1-KD
tubulin
55kDa

Fig 5A

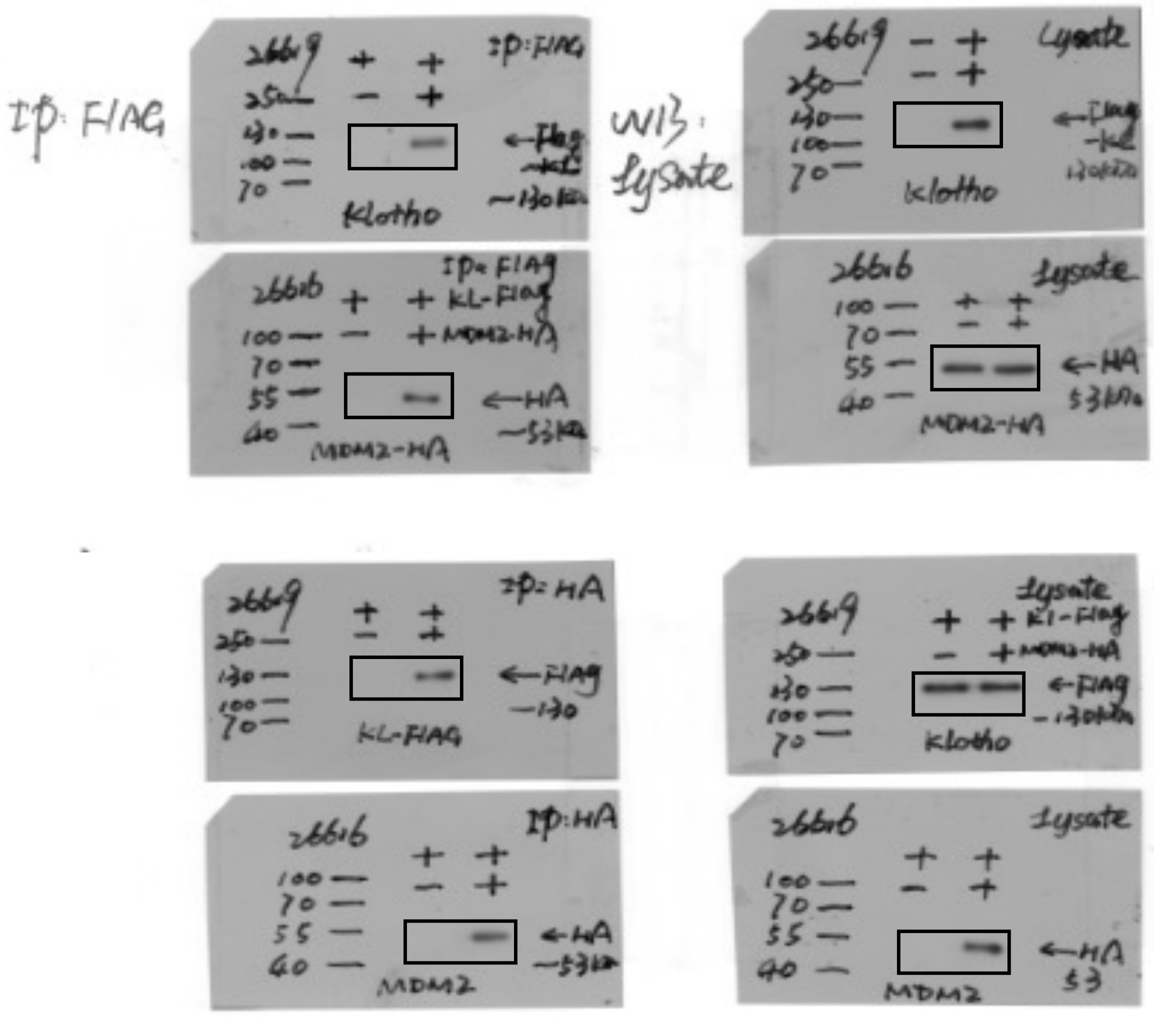
IP: FLAG
WB: lysate
IP:FLAG
Klotho
IP:FLAG
KL-Flag
MDM2-HA
MDM2-HA
Lysate
klotho
lysate
MDM2-HA
IP:HA
KL-FLAG
IP:HA
MDM2
lysate
KL-Flag
MDM2-HA
klotho
lysate
MDM2

Fig 5C

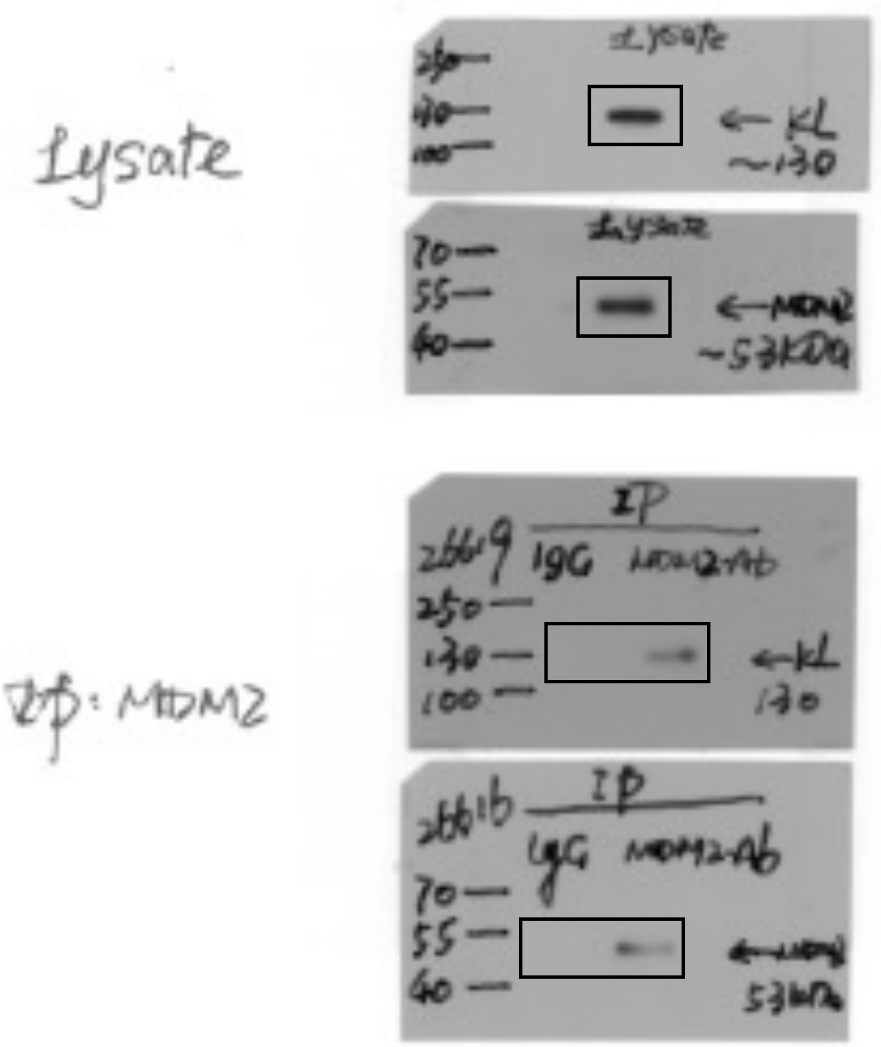
Lysate
Lysate
250
130
100
← KL
~130
Lysate
70
55
40
← MDM2
~53KDa
IP
IgG
130
100
← KL
130
IP
IgG
MDM2 Ab
70
55
40
53kDa

Fig 5D

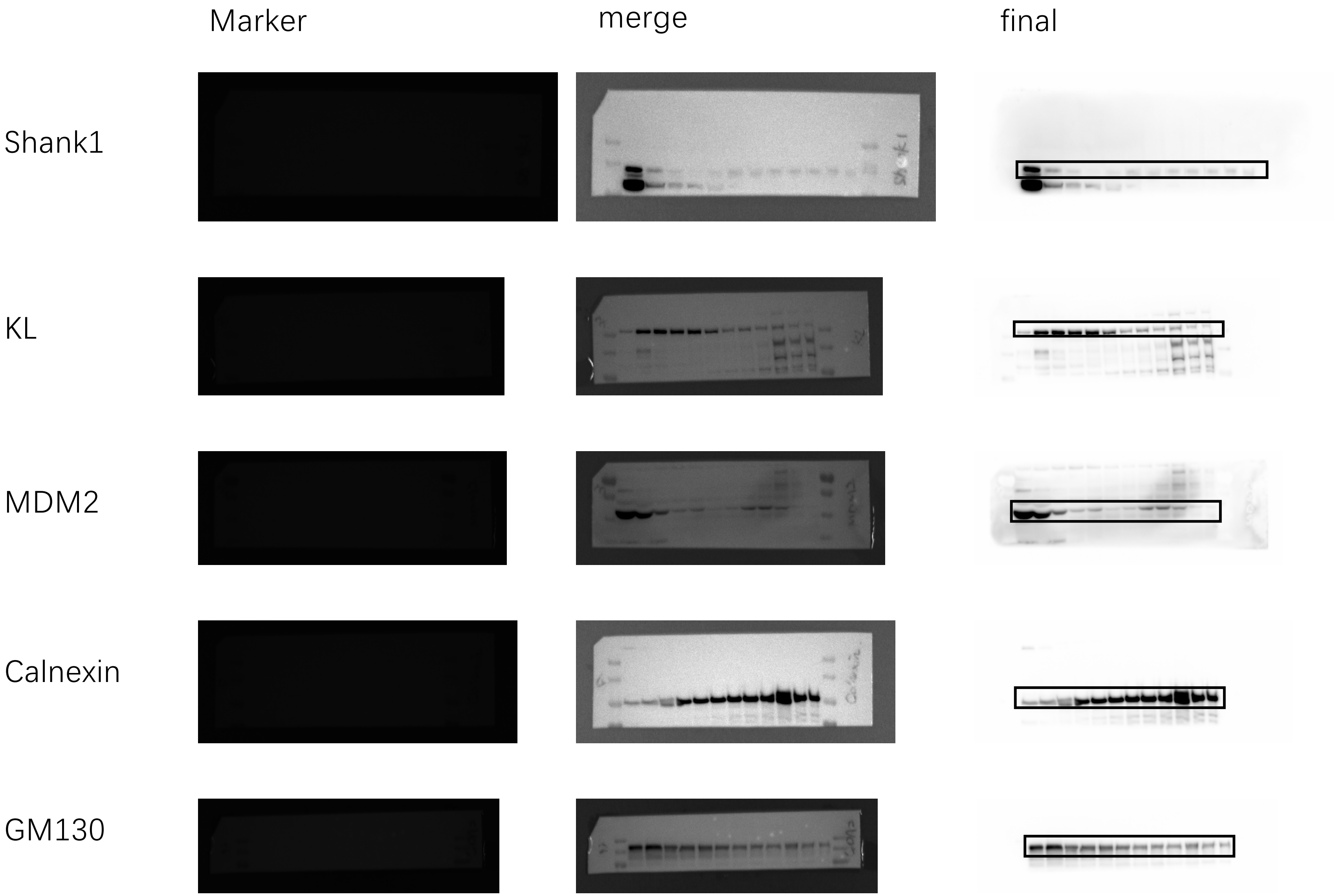

Fig 5E

Fig. 5G

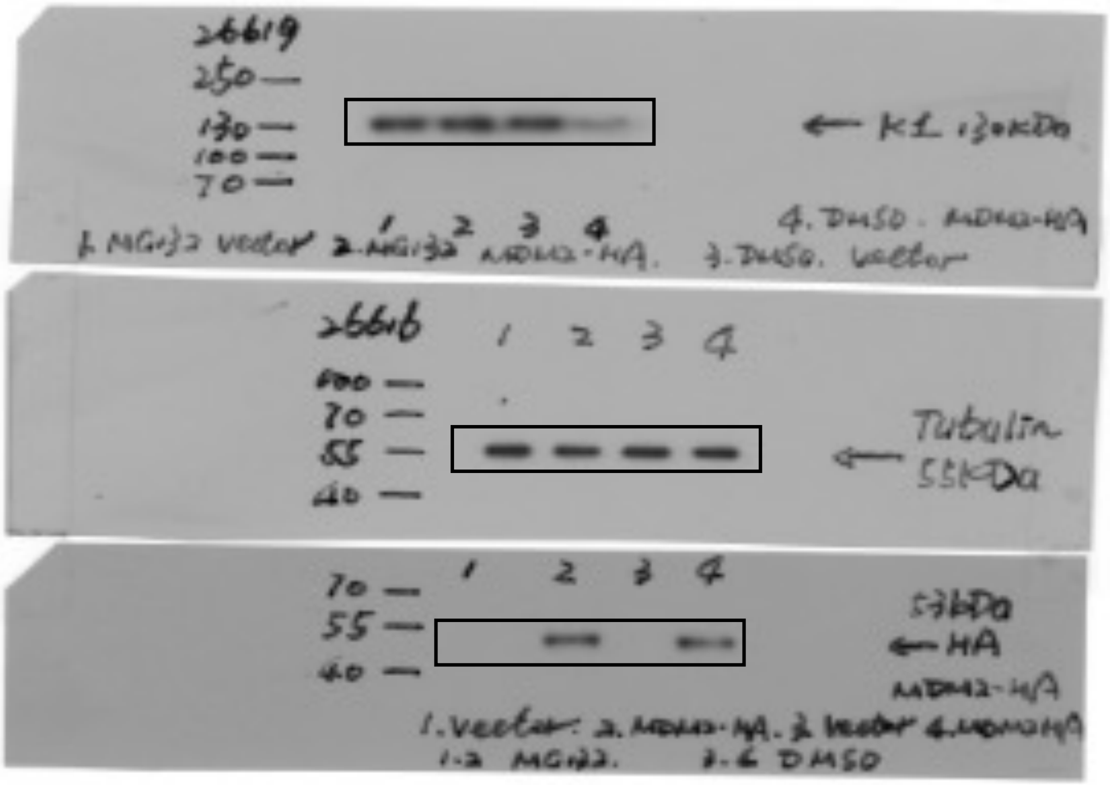

Fig 5G

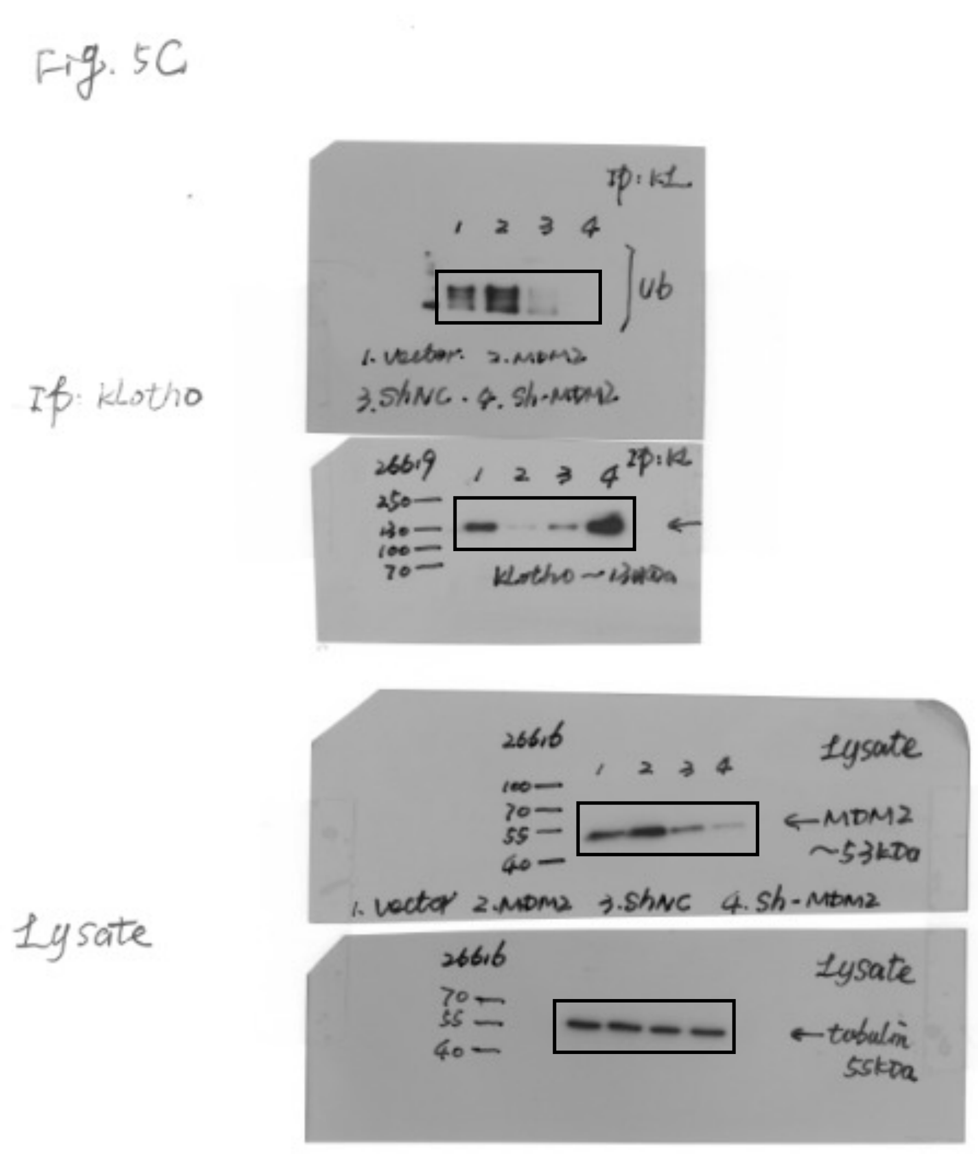
Fig. 5C
IP: klotho
IP: KL
1 2 3 4
Ub
1. vector 2. MDM2
3. ShNC 4. Sh-MDM2
26619
250
130
100
70
klotho ~130kDa
Lysate
26616
100
70
55
40
MDM2
~53kDa
1. vector 2. MDM2 3. ShNC 4. Sh-MDM2
tubulin
55kDa

Fig 6A

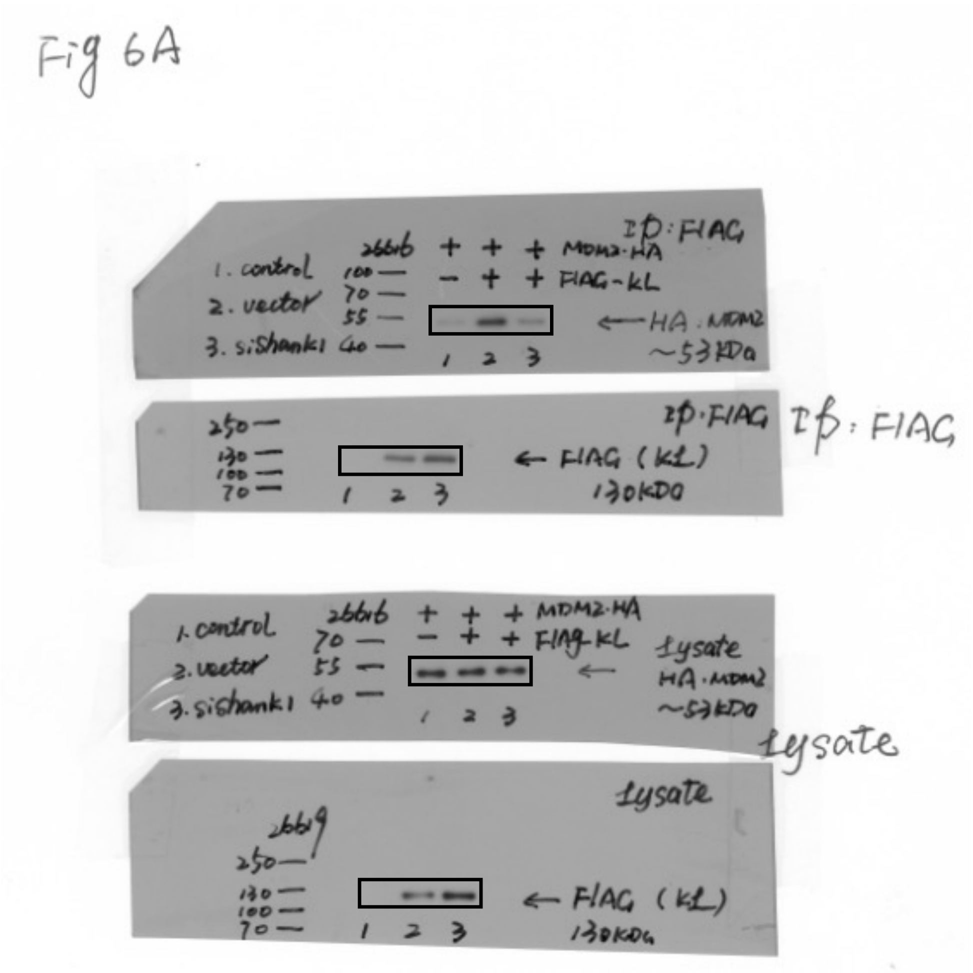
Fig 6A
IP: FlAG
1. control
2. vector
3. sishank1
MDM2-HA
FlAG-kL
← HA-MDM2
~53KDa
← FlAG (kL)
130KDa
lysate

Fig 6B

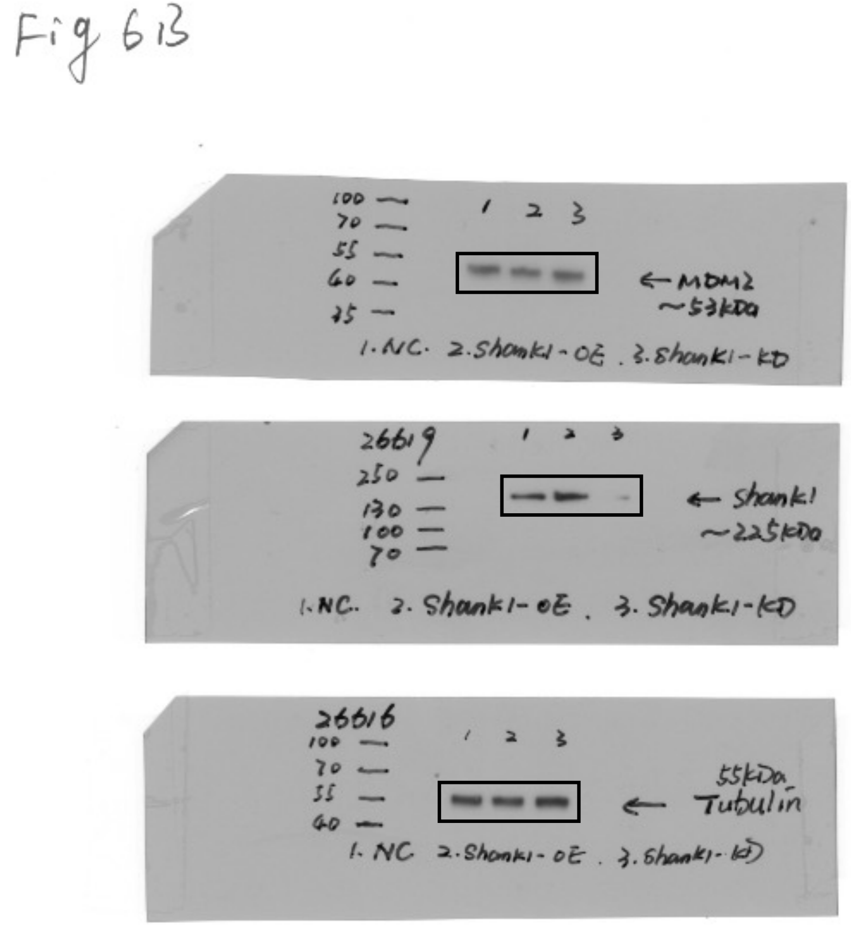

Fig 6B
100
70
55
40
35
1 2 3
← MDM2
~53kDa
1.NC. 2.Shank1-OE. 3.Shank1-KD
26619
250
130
100
70
1 2 3
← Shank1
~225kDa
1.NC. 2.Shank1-OE. 3.Shank1-KD
26616
100
70
55
40
1 2 3
55kDa
← Tubulin
1.NC 2.Shank1-OE. 3.Shank1-KD

Fig 6C

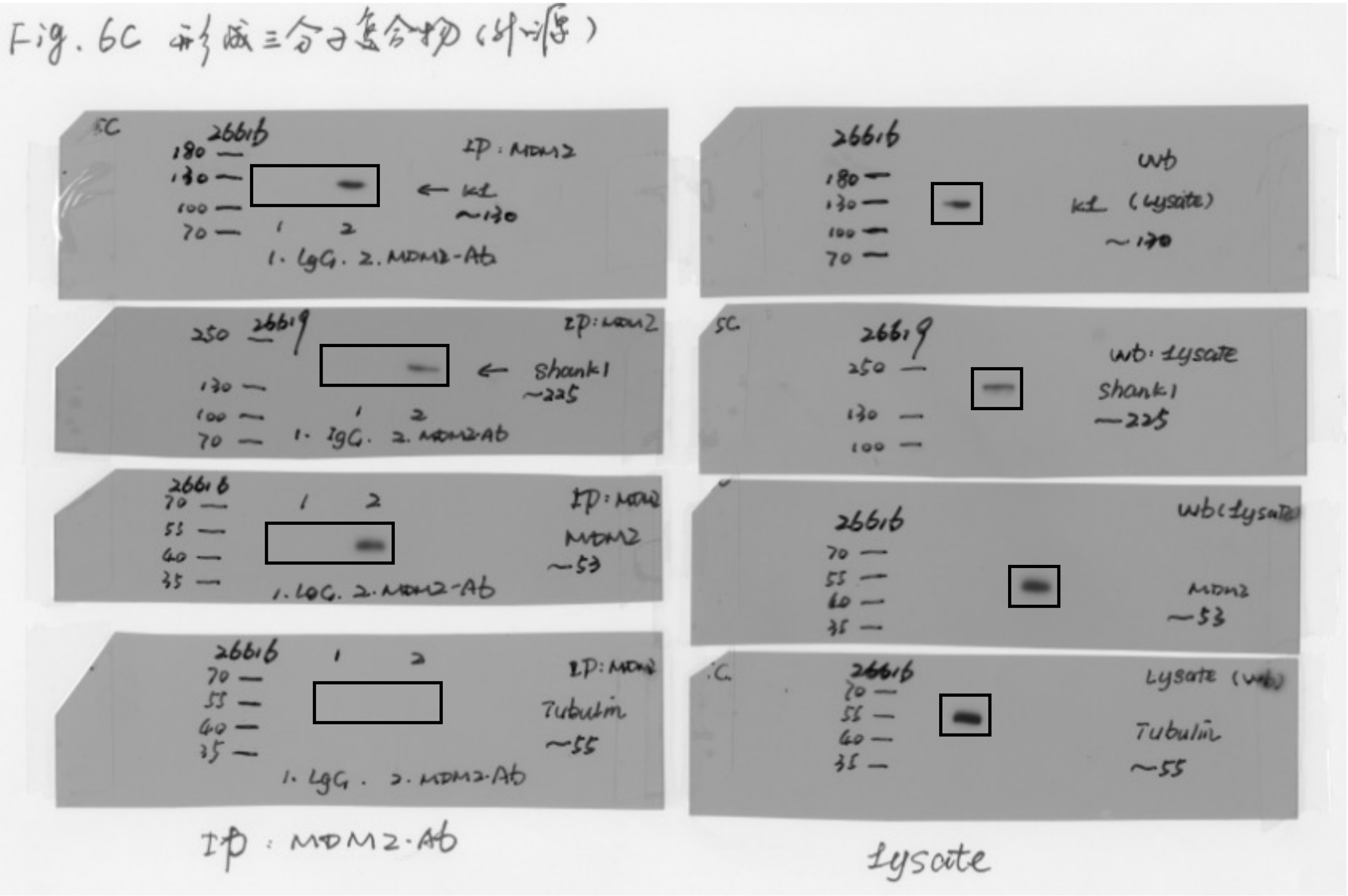

Fig 6D

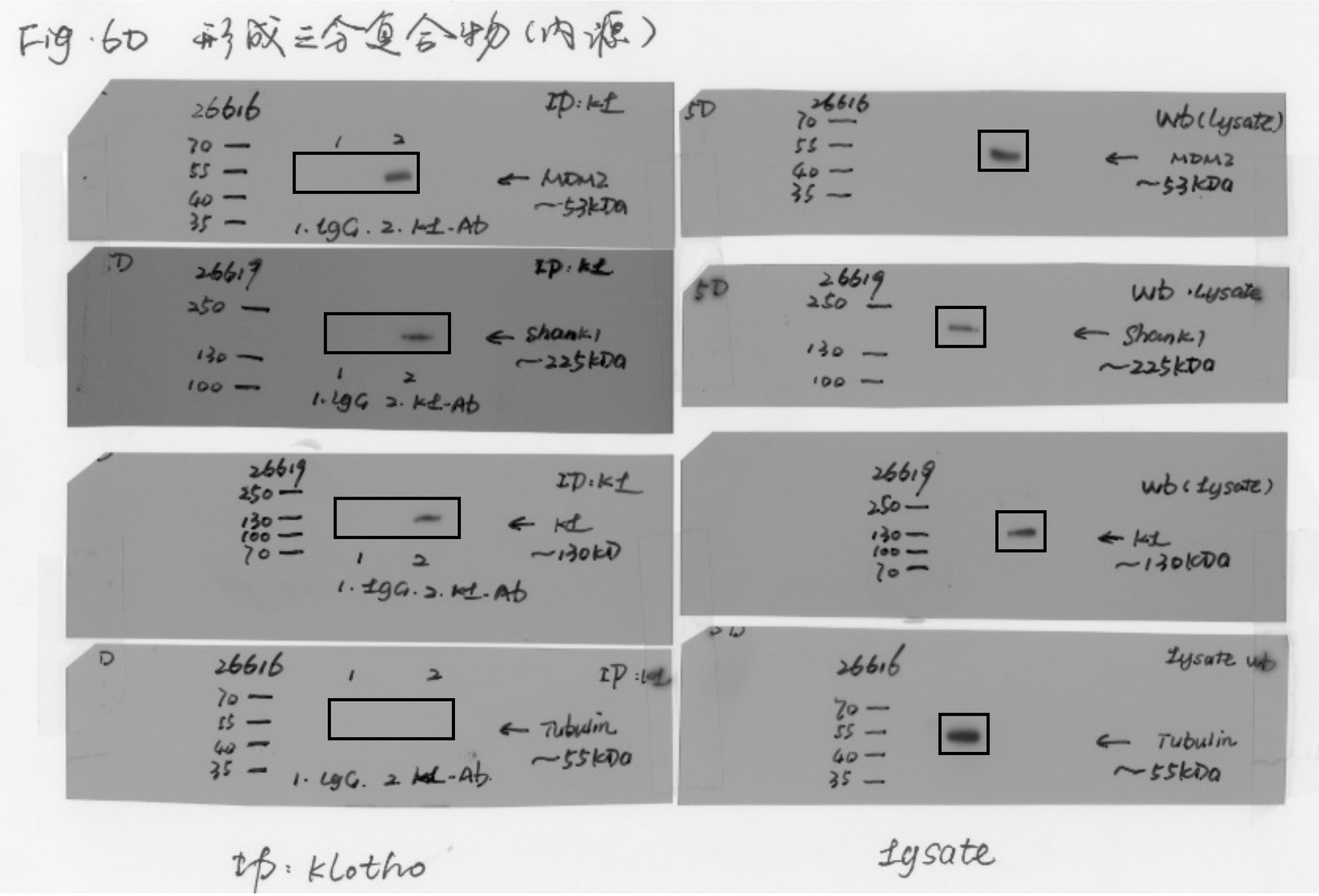
IP: kl
MDM2
~53kDa
1. IgG. 2. kl-Ab
Shank1
~225kDa
kl
~130kD
Tubulin
~55kDa
Wb (lysate)
~130kDa
lysate

Fig 6E， F

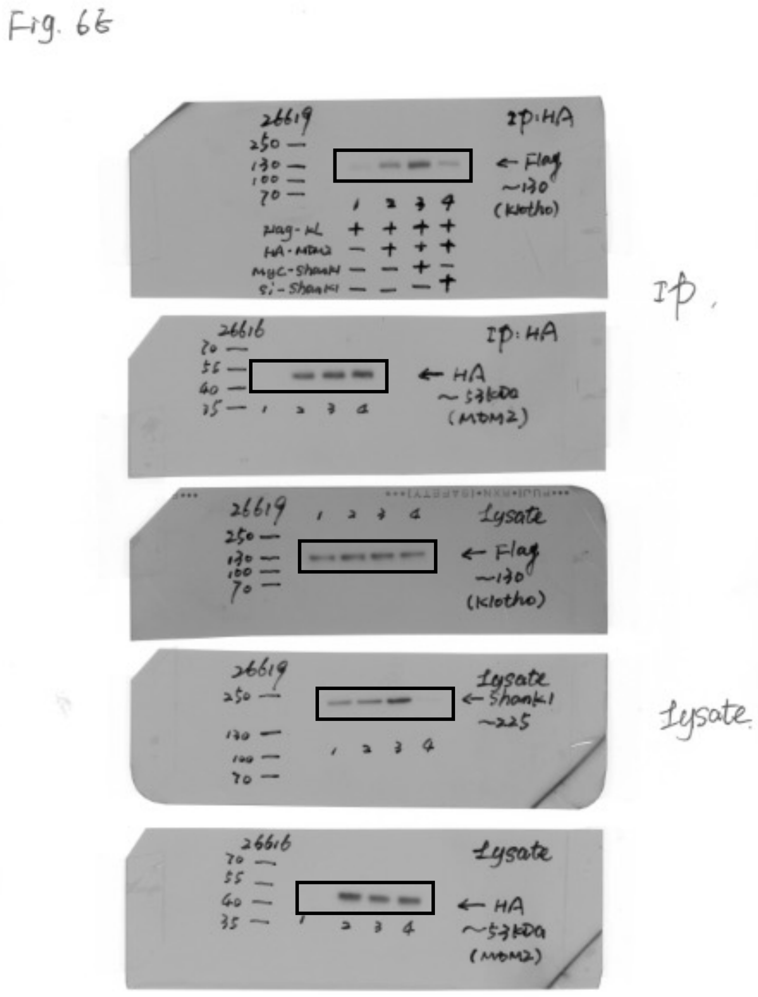
Fig. 6E
IP:HA
← Flag
~130
(klotho)
← HA
~53kDa
(MDM2)
Lysate
← Shank1
~225
IP.
Lysate

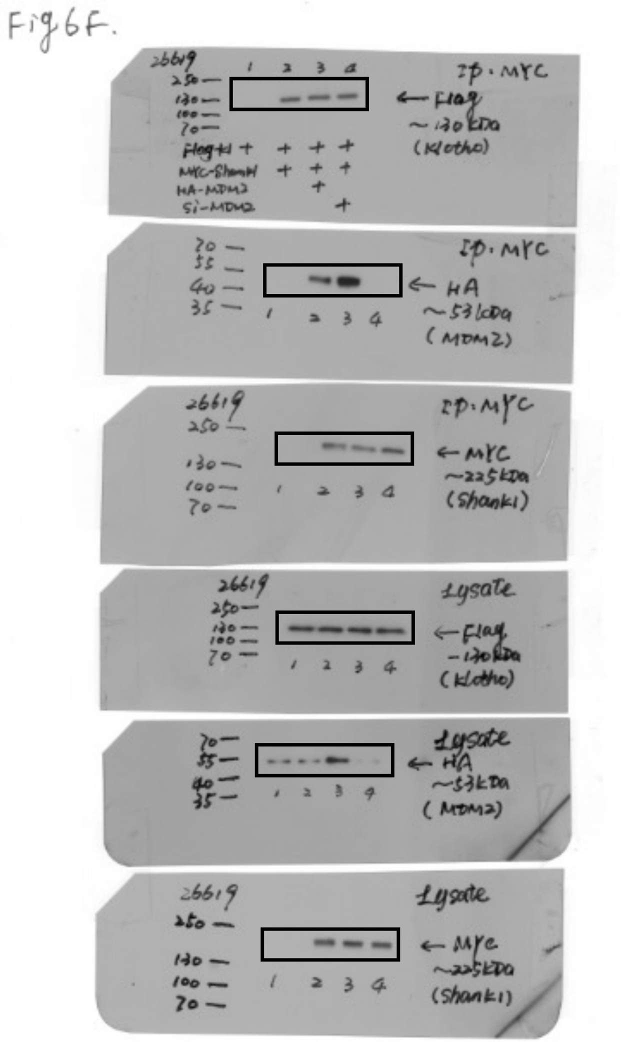
Fig6F.
IP: MYC
← Flag
~130kDa
(klotho)
← HA
~53kDa
(MDM2)
← MYC
~225kDa
(Shank1)
Lysate

Fig 6G，H

A549

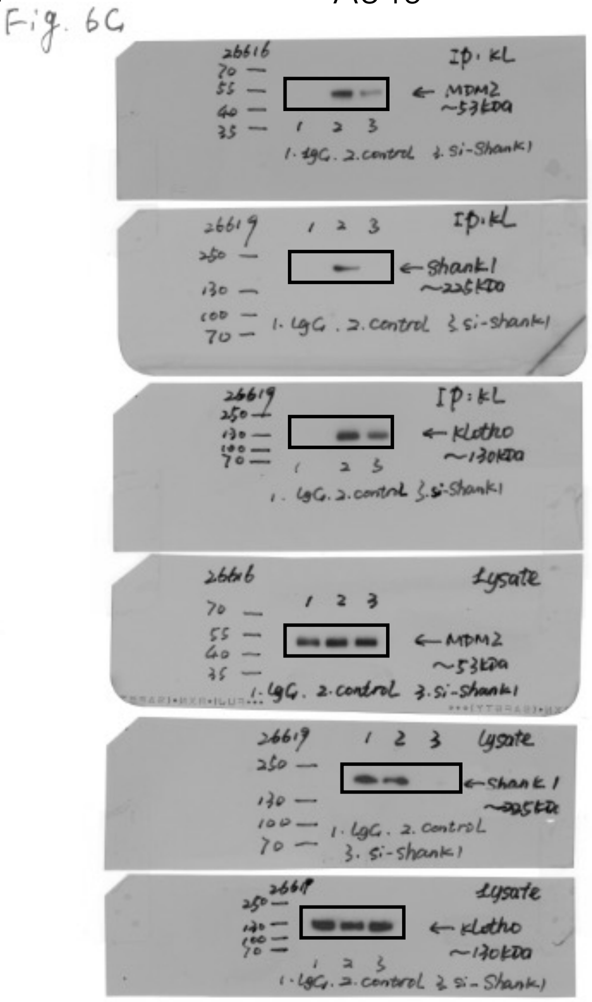

H1299

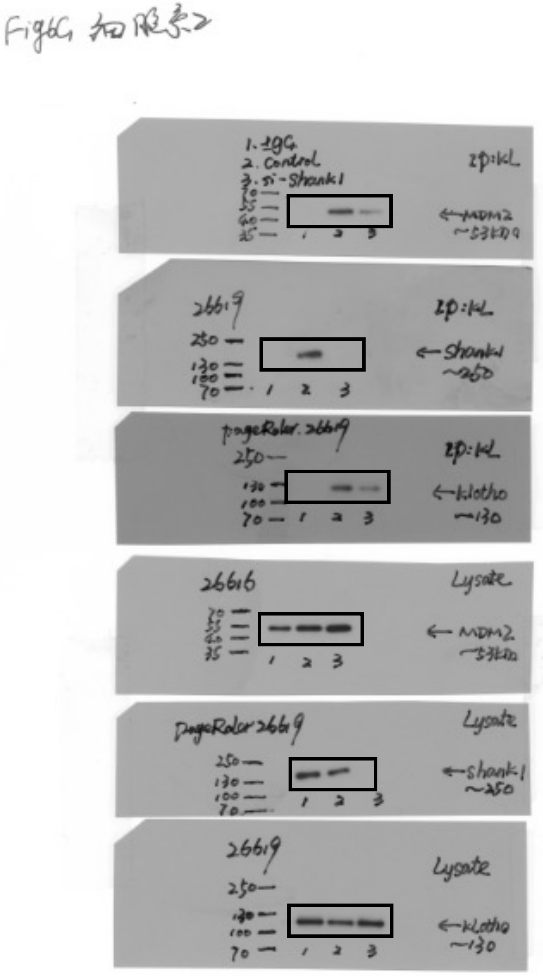

Fig 6I， J

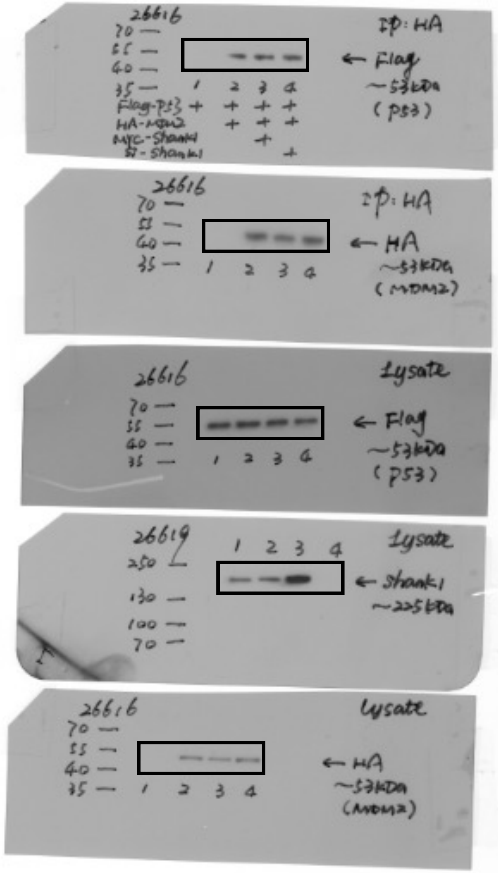

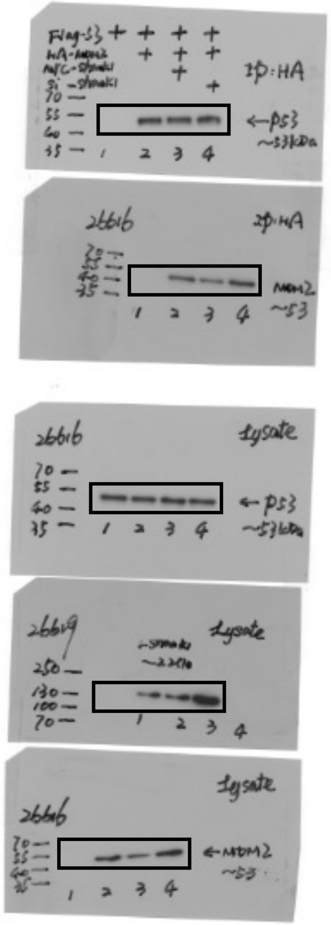

Fig 6K

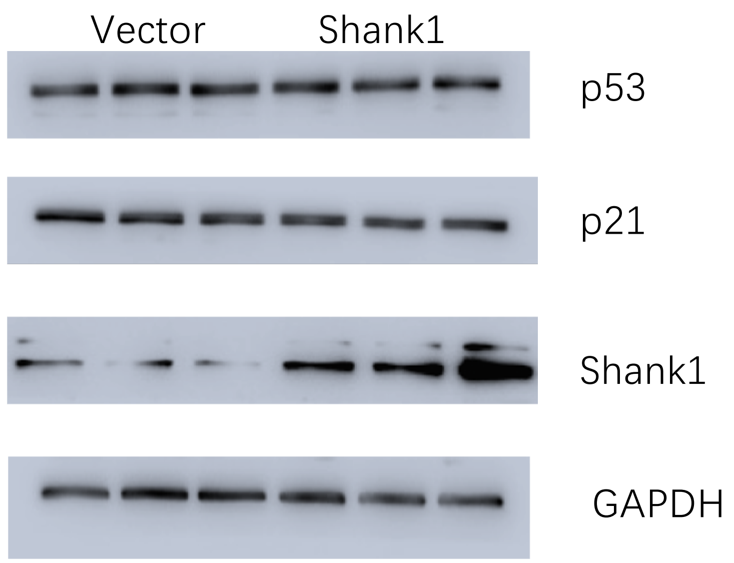

Fig 7D

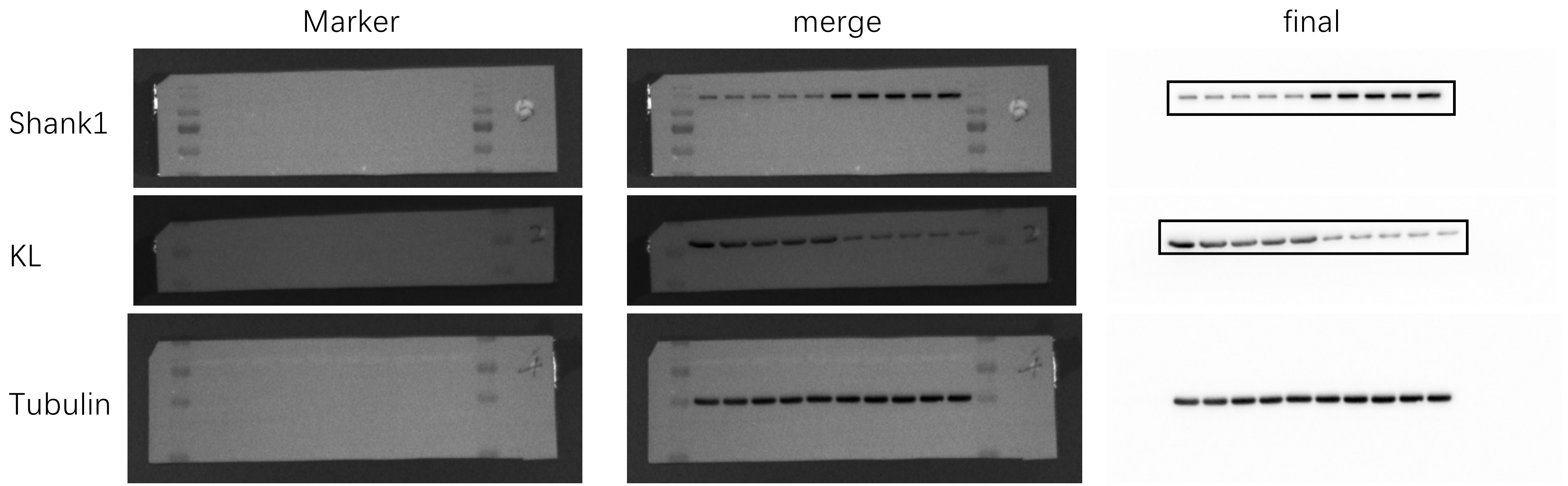

Fig 7D

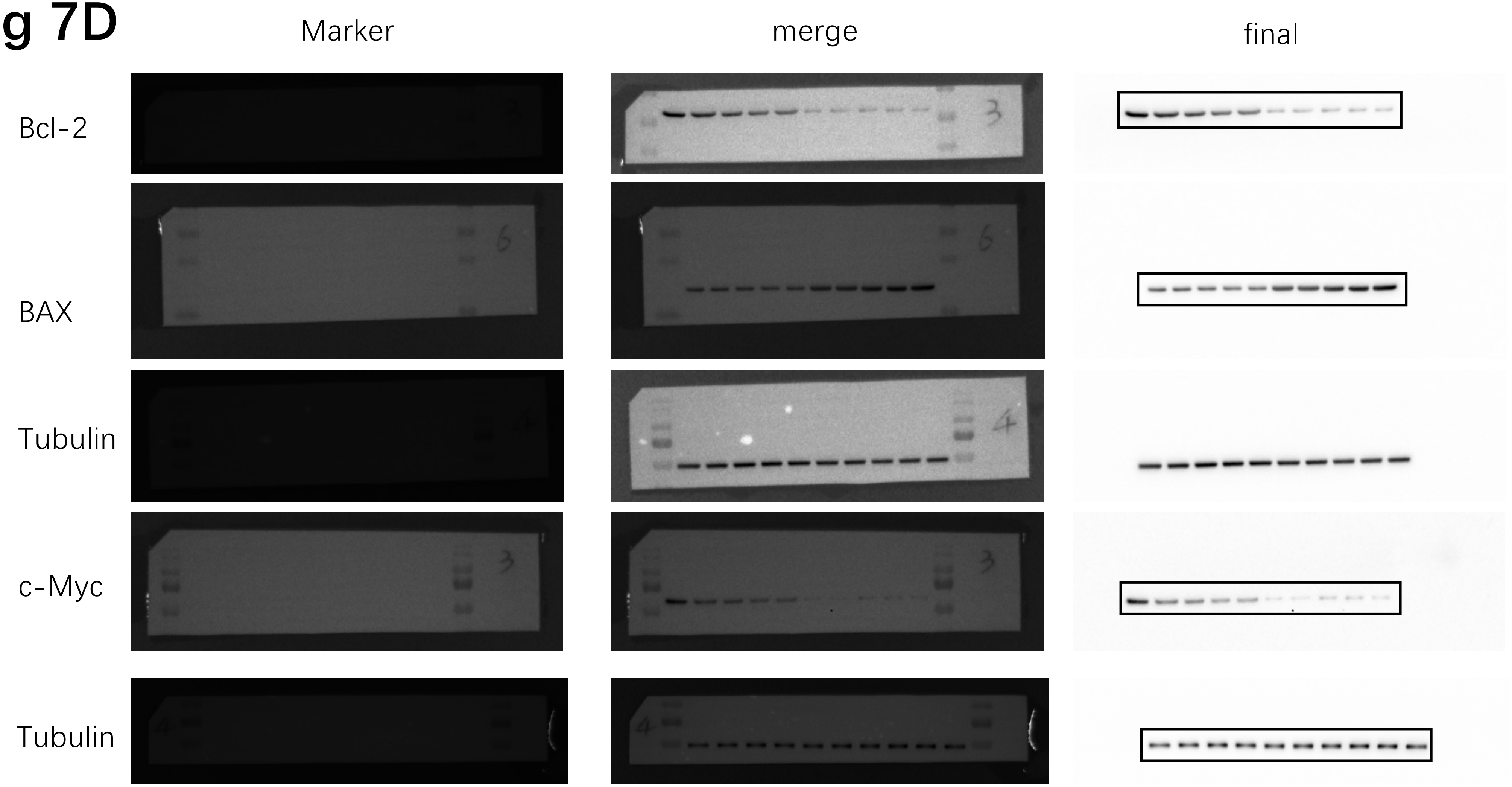

Supplement: Supplementary file 1 — Original western blots [file 41419_2022_4860_MOESM1_ESM.pdf]
